# Phylogenetic Constraints Do Not Explain the Rarity of Nitrogen-Fixing Trees in Late-Successional Temperate Forests

**DOI:** 10.1371/journal.pone.0012056

**Published:** 2010-08-06

**Authors:** Duncan N. L. Menge, Jeanne L. DeNoyer, Jeremy W. Lichstein

**Affiliations:** 1 National Center for Ecological Analysis and Synthesis, Santa Barbara, California, United States of America; 2 Department of Ecology and Evolutionary Biology, Princeton University, Princeton, New Jersey, United States of America; University of Leipzig, Germany

## Abstract

**Background:**

Symbiotic nitrogen (N)-fixing trees are rare in late-successional temperate forests, even though these forests are often N limited. Two hypotheses could explain this paradox. The ‘phylogenetic constraints hypothesis’ states that no late-successional tree taxa in temperate forests belong to clades that are predisposed to N fixation. Conversely, the ‘selective constraints hypothesis’ states that such taxa are present, but N-fixing symbioses would lower their fitness. Here we test the phylogenetic constraints hypothesis.

**Methodology/Principal Findings:**

Using U.S. forest inventory data, we derived successional indices related to shade tolerance and stand age for N-fixing trees, non-fixing trees in the ‘potentially N-fixing clade’ (smallest angiosperm clade that includes all N fixers), and non-fixing trees outside this clade. We then used phylogenetically independent contrasts (PICs) to test for associations between these successional indices and N fixation. Four results stand out from our analysis of U.S. trees. First, N fixers are less shade-tolerant than non-fixers both inside and outside of the potentially N-fixing clade. Second, N fixers tend to occur in younger stands in a given geographical region than non-fixers both inside and outside of the potentially N-fixing clade. Third, the potentially N-fixing clade contains numerous late-successional non-fixers. Fourth, although the N fixation trait is evolutionarily conserved, the successional traits are relatively labile.

**Conclusions/Significance:**

These results suggest that selective constraints, not phylogenetic constraints, explain the rarity of late-successional N-fixing trees in temperate forests. Because N-fixing trees could overcome N limitation to net primary production if they were abundant, this study helps to understand the maintenance of N limitation in temperate forests, and therefore the capacity of this biome to sequester carbon.

## Introduction

Symbiotic nitrogen (N) fixation is the most important N input to many ecosystems. It can be very productive—exceeding 50 kg N ha^−1^ y^−1^ in some temperate ecosystems [e.g., 1]—and it is the only natural N input that could feed back to plant N demand such that N neither limits primary production nor appears in excess. Nitrogen fixation (we hereafter drop the term ‘symbiotic’ since all N fixation we discuss here is symbiotic) is also at the heart of one of the most intriguing patterns in ecosystem ecology. Late-successional temperate forests (which we define as forests in which all individuals belonging to the initial cohort of trees have died) are often N-limited, yet ‘N fixers’ (here, tree taxa that have been shown to form N-fixing symbioses, or an individual tree that belongs to such a taxon, regardless of whether it is actively fixing N) are rare or absent in these forests [2]. Early in succession N limitation makes sense because available N is scarce but light, space, and other nutrients are relatively plentiful [3]. Accordingly, woody N fixers often dominate early-successional temperate forests [4]. However, it is generally thought that these woody N fixers are excluded during the course of succession, and that no N fixers are late-successional dominants in temperate forests [5].

**Figure 1:**
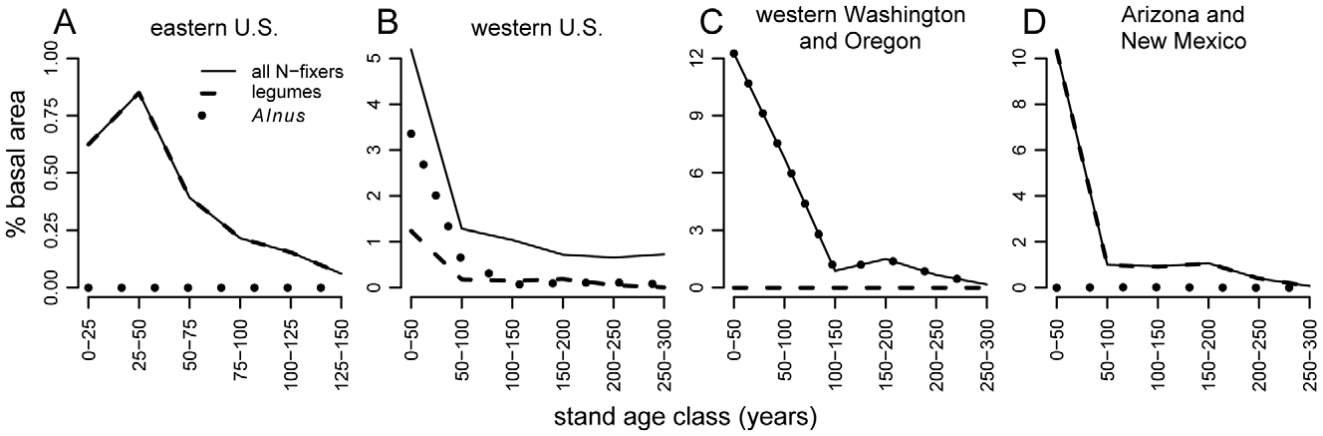
Mean percent basal area of N fixers vs. stand age. (A) Eastern and (B) western U.S. (coterminous U.S. east and west of 100° W longitude, respectively), (C) western Washington and Oregon (all counties west of the crest of the Cascade Mountains), and (D) Arizona and New Mexico. Basal area was calculated using FIA data, with 107,705; 28,454; 793; and 8,274 plot records, respectively, for panels A, B, C, and D. The dashed and dotted lines in B do not sum to the solid line because additional actinorhizal N-fixing taxa (e.g., *Cercocarpus* spp.) are present in the western U.S. (see Table S1).

**Figure 2:**
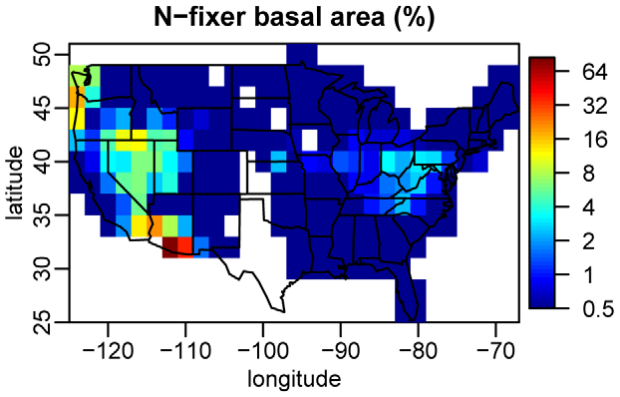
Geographical pattern of N fixer abundance. Grid cells are 2° latitude by 2° longitude. Values are the percentage of total basal area in each grid cell comprised by N fixers. Note the logarithmic color scale. All grid cells with a value <0.5% were assigned the value 0.5% in the map. White spaces reflect grid cells with fewer than 20 FIA plots.

Data from a systematic inventory of forests and woodlands in the coterminous U.S. (i.e., excluding Alaska and Hawaii) are consistent with the conjecture that N fixer abundance in temperate forests is highest early in succession and declines with stand age (Fig. 1, see Methods for details). The successional decrease in N fixer abundance is unmistakable and geographically consistent (Fig. 1), although the species composition (Figs. 1, S1, Table S1) and total abundance (Figs. 1, 2, Table S1) of N fixers vary geographically. In contrast to late-successional temperate forests, N fixers are common in many other ecosystems, including savannas [6], grasslands [7], [8], tropical forests [5], [9], and chaparral [10], [11], [12]. For example, among 50-ha plots in the Center for Tropical Forest Science tropical forest network, legumes (many of which are N fixers) comprise 6–15% of basal area in the neotropics, 9–74% in Africa, and <2–9% in Asia [9]. Although mean N fixer abundance in younger U.S. forests (up to 12% of basal area; Fig. 1) rivals some of the tropical sites, it is low in older U.S. forests (<1%; Fig. 1). This rarity, combined with the fact that N limitation prevails in many late-successional temperate forests [2], [13], [14], presents an intriguing paradox with important implications for carbon and N cycling [2], [15].

There are two classes of explanation for the rarity of N fixers in late-successional temperate forests. First, phylogenetic constraints may prevent N fixation from evolving in late-successional taxa or late-successional traits from evolving in N fixers. For example, if all N fixers were in a phylogenetic clade whose members (both N fixers and non-fixers) all had early-successional traits, then no late-successional taxa would be genetically predisposed to evolving N fixation, and no N fixers would be genetically predisposed to being late successional. Put another way, the phylogenetic constraints hypothesis says that N fixers are rare in late-successional temperate forests simply because there have been no taxa with the genetic material to both fix N and be late successional. Second, selective constraints such as physiological or ecological tradeoffs inherent to N fixation may restrict N fixer abundance in late-successional temperate forests. For example, N fixers may be poorer competitors for soil resources because additional fine roots could have been constructed instead of root nodules [16], [17]. Numerous viable aspects of the selective constraints hypotheses have been proposed [e.g.], [ 16], [17], [18], [19,20] (see Discussion), but the phylogenetic constraints hypothesis has not to our knowledge been tested. In the absence of such a test, the practical significance of the growing literature on selective constraints is uncertain [21].

It has been argued that phylogenetic constraints might preclude N fixation in temperate forests because woody legumes are rare outside the tropics [21]. However, there are some widespread woody legumes in temperate forests [e.g., *Robinia pseudoacacia* (black locust), *Cercis canadensis* (redbud); see Table S2]. Furthermore, not all N fixers are legumes [22], and not all legumes are N fixers [23], [24]. All root-nodulating N fixers worldwide reside in a monophyletic subclade of the Eurosid I clade [25], which contains legumes as well as hundreds of woody actinorhizal (non-leguminous) N-fixing species spread across 25 genera from eight families [22]. Many woody actinorhizal species occur in temperate biomes, and although they form symbioses with different bacteria than legumes do, the N fixation enzyme is the same. Thus, the rarity of woody N-fixing species in late-successional temperate forests is not because of a restricted geographical range. Whether or not phylogenetic constraints prevent these N fixers from being abundant late in succession, therefore, depends on the phylogenetic relationship between the N fixation trait and traits associated with being late successional.

In the present work we evaluate the common but untested assumption that N-fixing tree taxa in temperate forests are more early successional than non-fixing tree taxa, and we assess the viability of the phylogenetic constraints hypothesis as an explanation for this pattern. Specifically, we use phylogenetically independent contrasts (PICs) to test for evolutionary relationships between N fixation and indices of successional status. These indices quantify differences among taxa in terms of shade tolerance and the age of forest stands in which they tend to occur (see Methods for details). Significantly negative evolutionary relationships between N fixation and the successional indices would mean that N fixers are more early successional than their non-fixing close relatives. The existence of late-successional non-fixing close relatives would suggest that late-successional N fixers were feasible in terms of evolutionary history, thereby refuting the phylogenetic constraints hypothesis. Additionally, negative evolutionary relationships would imply concurrent evolutionary change in N fixation and successional status, suggesting that selection has acted against the combination of N fixation and late-successional traits. Therefore, in addition to rejecting the phylogenetic constraints hypothesis, negative evolutionary relationships would support the selective constraints hypothesis. We also quantify the phylogenetic signals of N fixation and each successional index, which indicate the evolutionary conservatism of each trait. Conservative traits change slowly over evolutionary time, so a lack of conservatism in N fixation or the successional traits would further contradict the phylogenetic constraints hypothesis.

### Phylogenetic context and definitions

**Figure 3:**
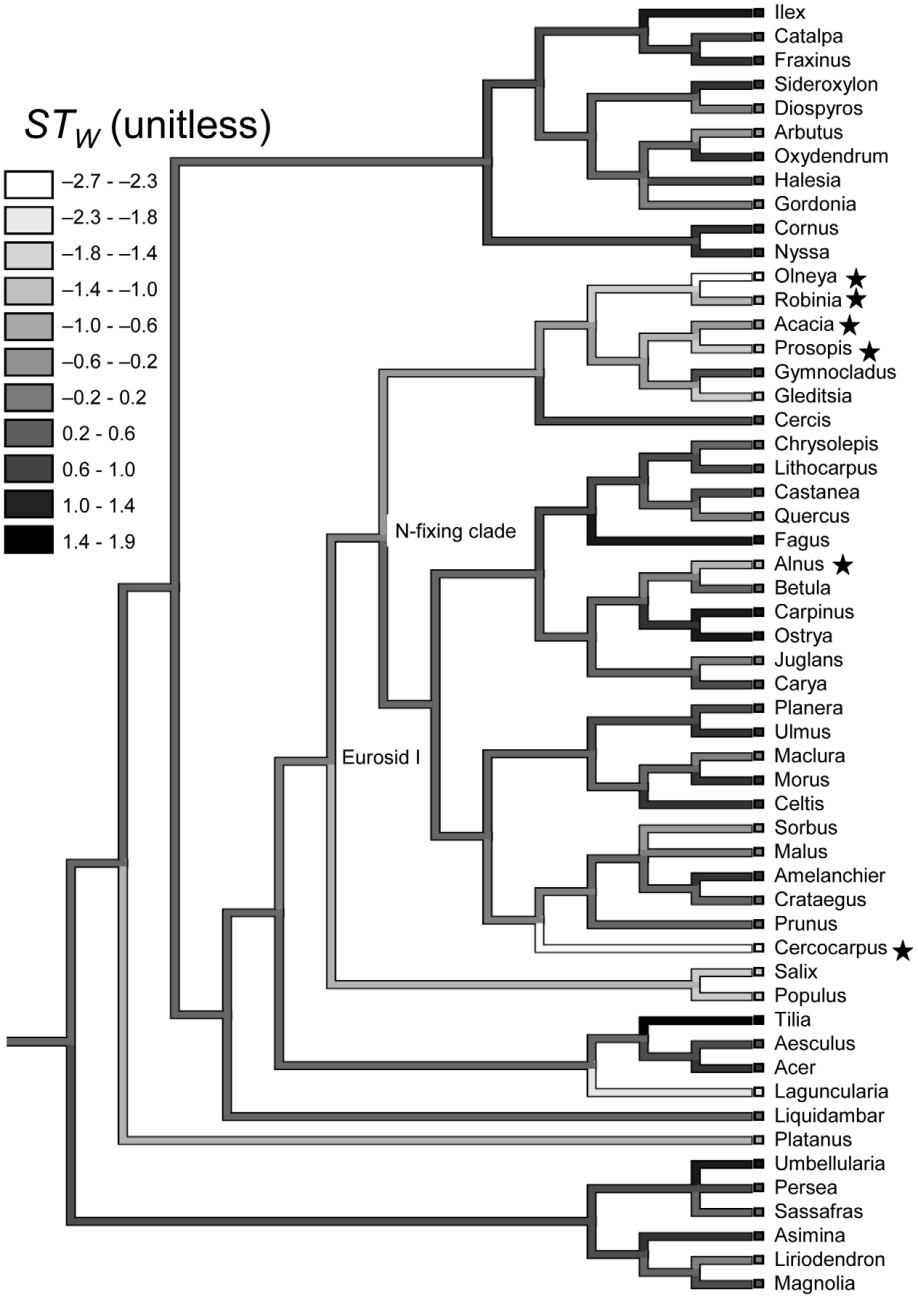
Character history reconstruction of the weighted shade-tolerance index (*ST_W_*) for native angiosperm FIA genera. The shading indicates the character state of *ST_W_*, with darker shading indicating greater shade tolerance. *ST_W_* is the proportion of a taxon's saplings in the FIA data that are in the understory relative to the mean shade tolerance across taxa in 2°×2° grid cells (Fig. S3), and is expressed as the number of standard deviations from the overall angiosperm mean (see Methods for details). The six genera that form N-fixing symbioses are starred: *Olneya*, *Robinia*, *Acacia*, and *Prosopis* (Fabaceae); *Cercocarpus* (Rosaceae); and *Alnus* (Betulaceae). Two clades are indicated: the Eurosid I clade and the ‘potentially N-fixing clade,’ the smallest clade that includes all N fixers (the monophyletic subclade of the Eurosid I that excludes the Malpighiales). To create this figure we used the Trace Character History function (parsimony method) of the program Mesquite [66].

All N fixers reside in a monophyletic subclade of the Eurosid I angiosperm clade [25] (Fig. 3). We define this subclade, the smallest clade that contains all N fixers, as the ‘potentially N-fixing clade’ (Fig. 3), and we define a ‘candidate N fixer’ as a non-fixer that is in the potentially N-fixing clade. To understand why we refer to non-fixers in the potentially N-fixing clade as ‘candidate N fixers,’ consider the two plausible histories of the N fixation trait. (i) N fixation evolved once—presumably at the base of the potentially N-fixing clade—and has since been lost multiple times. Alternatively, (ii) N fixation evolved multiple times after the potentially N-fixing clade diversified, in which case all taxa in the potentially N-fixing clade likely possess some trait that facilitates the evolution of N fixation [25]. Current evidence suggests that the case of multiple origins (ii) is more likely [26]. If N fixation has indeed evolved many times in the potentially N-fixing clade, and not once outside this clade (ignoring loose associations that are not true symbioses, such as *Gunnera/Nostoc*; *Gunnera* is in the Eurosid I but not in the subclade), it is likely that plants in the potentially N-fixing clade are genetically predisposed to evolve N-fixing symbioses. Our main conclusions, however, do not depend on whether (i) or (ii) is the true history (see Discussion).

## Methods

### Forest Inventory and Analysis overview

We used data from the U.S. Forest Service's Forest Inventory and Analysis (FIA) database (http://www.fia.fs.fed.us/), version 2.1, to derive species- and genus-specific shade tolerance and stand age indices and to quantify successional and geographical patterns in N fixer basal area. FIA plots are located systematically across coterminous U.S. forests and woodlands (1 plot per ∼2,400 ha). Trees [diameter at breast height (dbh)≥12.7 cm] are measured on four 7.3 m radius subplots per plot, and saplings (dbh 2.54–12.7 cm) are measured on four 2.1 m radius subplots. To ascertain which genera and species in the FIA are N fixers, we used published reports of actinorhizal genera [22] and rhizobial species [23], [24] that have been shown to nodulate or fix N. Because the FIA is a systematic survey, any tree taxa not represented in this dataset are rare; thus, our analysis includes all common coterminous U.S. tree taxa. Shrubs and some small tree taxa are not sampled by the FIA.

### Successional indices

We defined successional indices related to shade tolerance and stand age that could be calculated for each taxon (genus or species) in the FIA dataset. Unlike categorical shade tolerance classifications [e.g., 27], our indices can be calculated at any taxonomic level and can be objectively quantified from widely available inventory data. We calculated multiple indices to evaluate the robustness of our results. Our shade tolerance index (*ST_U_*; *U* for “unweighted”) is the proportion of live saplings of a taxon that is in the understory (as opposed to the canopy; see details below). Shade-tolerant taxa with high understory survivorship [28], [29] are more likely to persist in the understory than shade-intolerant taxa [30], [31] and should therefore have relatively high values of *ST_U_*. Species with high *ST_U_* tend to have high survival rates and low growth rates [31], as is typical of late-successional species [32], [33]. Furthermore, *ST_U_* is strongly correlated with a widely used categorical shade tolerance classification (Fig. S2). Our stand age index (*SA_U_*) is the mean age of the stands in which a taxon occurs (see details below), so by definition it is related to successional status.

Interpreting *ST_U_* and *SA_U_* is not completely straightforward because both indices vary geographically within the U.S. For example, taxa tend to have relatively low *ST_U_* in the southwestern U.S. (Fig. S3C), a semi-arid to arid region characterized by open woodlands and deserts; and taxa tend to have relatively high *SA_U_* in the western U.S., where forests tend to be older than in the eastern U.S. (Fig. S3A,B). To account for geographical variation in *ST_U_* and *SA_U_*, we calculated geographically weighted versions of the indices.

Shade tolerance indices were calculated using the FIA's ‘crown class’ (overtopped, intermediate, co-dominant, dominant, or open-grown), which is reported for live trees and saplings. We considered saplings with an overtopped or intermediate crown class to be in the understory. The geographically weighted index, *ST_W_*, quantifies the shade tolerance of each taxon relative to other taxa in each 2° latitude by 2° longitude grid cell. For each taxon *ST_W_* is
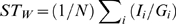
(1)where *N* is the number of live saplings in the taxon with a reported crown class, the summation is over *N*, *I_i_* is 1 if sapling *i* is in the understory and 0 otherwise, and *G_i_* is the proportion of saplings (all taxa combined) in sapling *i*'s grid cell that are in the understory (‘overtopped’ or ‘intermediate’ FIA crown class). Taxa with low values of *ST_U_* (shade-intolerant taxa in absolute terms) may have high values of *ST_W_* if they are more shade tolerant than co-occurring taxa. The *ST_W_* values we present are normalized within angiosperms to have zero mean and unit variance. Values near zero indicate taxa that have similar shade tolerance to their geographical neighbors, while positive and negative values, respectively, indicate greater and lesser shade tolerance than neighboring taxa. We report *ST_W_* for all native angiosperm taxa with at least 20 live saplings with a reported crown class in the FIA dataset (166 species, 54 genera; Table S2).

Stand age indices were based on reported stand age for each FIA plot, which FIA defines as “the average age of the live trees not overtopped in the predominant stand size-class” and is estimated by coring several trees [34]. Thus defined, stand age typically increases monotonically with time since the last stand-replacing disturbance [35]. We calculated geographically weighted versions of stand age based on both the mean and maximum stand age in each 2°×2° grid cell. For each taxon, we calculated *SA_W-mean_* as
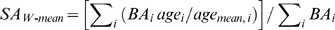
(2)and *SA_W-max_* as
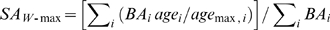
(3)where *BA_i_* is the basal area of individual (tree or sapling) *i*, *age_i_* is the age of the plot (in years) in which individual *i* resides, *age_mean,i_* and *age_max,i_* are the mean and maximum stand age of plots in individual *i*'s grid cell (Fig. S3), respectively, and the summations are over all individuals in the taxon in plots with a reported stand age. As with *ST_W_*, we report *SA_W-mean_* and *SA_W-max_* values that are normalized within angiosperms to have mean 0 and variance 1. *SA_W-mean_* and *SA_W-max_* have similar interpretations, but the exact values depend on geographic variation in disturbance history (both natural and anthropogenic). We report the stand age indices for all native angiosperm taxa with at least 20 live individuals (trees or saplings) in a plot with a reported stand age in the FIA dataset (189 species and 54 genera; Table S2).

We present results based on the weighted indices (*ST_W_*, *SA_W-mean_*, and *SA_W-max_*) in the main text. The unweighted indices (*ST_U_* and *SA_U_*) are reported in Table S2. We restricted our analyses to native species because our evolutionary questions are best addressed by analyzing species that have evolved in their current range. This restriction excludes one exotic N fixer from our *ST* analyses—*Elaeagnus angustifolia* (Russian olive), an invasive [36], [37] shrub-tree in the Elaeagnaceae—and two from our *SA* analyses—*E. angustifolia* and *Albizia julibrissin*, a tree in the Fabaceae. Including introduced species into our analyses does not qualitatively change our results (results not shown).

### Phylogeny of FIA angiosperms

We constructed a genus-level phylogeny for all native genera for which successional indices were available. We used a genus-level phylogeny because there is little topological resolution for species and because most N-fixing genera contain only N-fixing species. As a starting point, we downloaded the newick code for the family level and some of the genus level phylogenetic topology from the Phylomatic website (http://svn.phylodiversity.net/tot/megatrees/R20091120.new). This tree is based in part on the latest publication of the Angiosperm Phylogeny Group, APG-III [38]. We added genus level resolution to the Fagaceae using a published phylogeny for this group [39] and added branch lengths (in millions of years; used to calculate independent contrasts) to this tree using the bladj command in Phylocom [40] and published fossil ages of some of the internal nodes [41]. The bladj command uses these fossil ages to interpolate the remaining node ages.

### Statistical analyses

Classical statistical tests, which assume independent errors, and thus ignore the evolutionary relationships among taxa, may yield biased results in comparative analyses. PICs correct for the lack of independence among related taxa [42]. We used the Analysis of Traits module in Phylocom [40] to calculate PICs of and to test for evolutionary relationships between N fixation and each successional index for FIA angiosperm genera. We performed four separate analyses with the PICs for each successional index, one for each combination of phylogenetic extent (all FIA angiosperms or just the potentially N-fixing clade) and treatment of the N fixation trait (discrete vs. continuous). With N fixation modeled discretely, Phylocom treats each genus as an N fixer or a non-fixer (1 or 0) and calculates the unstandardized PICs of each successional index only on those nodes for which contrasting states of N fixation occur on at least two of the descendent nodes. Conversely, when N fixation is modeled continuously, Phylocom assigns to each genus the proportion of species in that genus that fix N and calculates *n*-1 PICs for each trait, where *n* is the number of internal nodes, standardized with the branch lengths of the phylogeny. Because some genera contain both N-fixing and non-fixing species [23], the continuous analysis—which allows for this possibility—seems more biologically reasonable. We also present the discrete case, which is statistically more conservative, because none of the N-fixing species in our analysis have non-fixing congeners.

We performed one-tailed *t*-tests of the alternative hypotheses that N fixers are more early successional (lower *ST* and *SA*) than non fixers. For the discrete case, we calculated one-sample *t*-statistics from the PIC means and standard deviations reported by Phylocom. For the continuous case, Phylocom calculates the Pearson correlation coefficient between the PICs for both traits, and we tested the significance of this correlation coefficient by converting it to a *t*-statistic [43]. We also used Phylocom to calculate the phylogenetic signal for each trait (N fixation and each successional index), i.e., the degree to which each trait is conserved across the phylogenies [40], [44], [45]. Phylocom tests for phylogenetic signal by comparing the PIC means of the observed tree to the distribution of PIC means from 1000 randomizations of trait values across the tips of a phylogeny. We used two-tailed tests for phylogenetic signal.

## Results

### N-fixing taxa

Among U.S. tree taxa with at least 20 live saplings in the FIA dataset (required for *ST* indices; Table S2), there were 9 N-fixing species in 6 genera and 3 families. There were 7 rhizobial species: *Acacia* spp. (acacias), *Olneya tesota* (desert ironwood), *Prosopis glandulosa*, var. *torreyana* (western honey mesquite), *Prosopis velutina* (velvet mesquite), *Prosopis* spp. (mesquite), *Robinia neomexicana* (New Mexico locust), and *Robinia pseudoacacia*, all in the Fabaceae; and 2 actinorhizal species: *Alnus rubra* in the Betulaceae and *Cercocarpus ledifolius* in the Rosaceae. The potentially N-fixing clade contained 54% of all genera and 62% of all species. For *SA* indices we included taxa with at least 20 live individuals (saplings or trees), which added an N-fixing species—*Alnus rhombifolia* in the Betulaceae—and changed the proportion of species in the potentially N-fixing clade to 61%.

### Shade tolerance and N fixation

**Figure 4:**
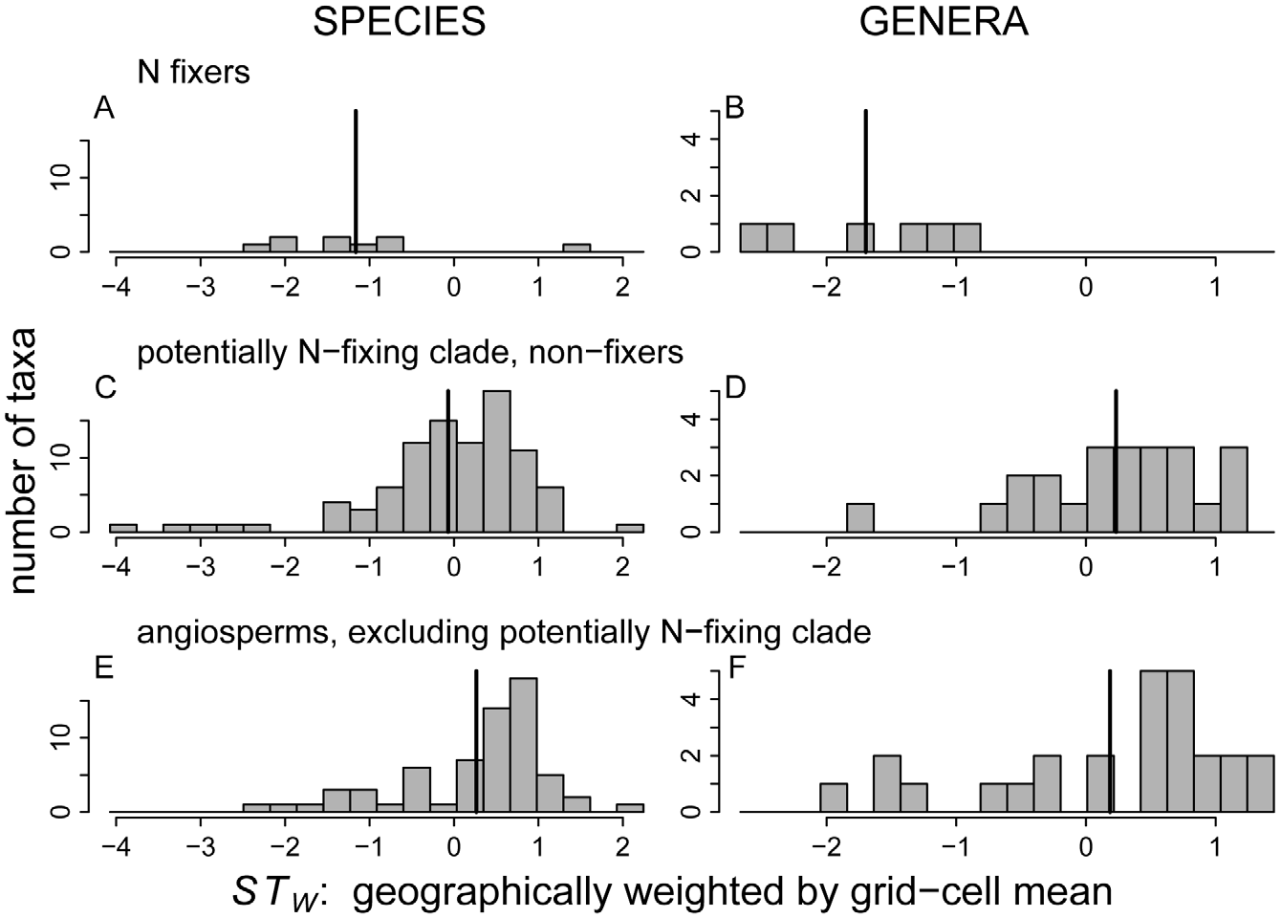
Histograms of the geographically weighted shade tolerance index (*ST_W_*) for native FIA angiosperm taxa. Both species (A, C, E) and genera (B, D, F) are shown. The data are divided into (A, B) N fixers, (C, D) non-fixers in the potentially N-fixing clade, and (E, F) non-fixers outside the potentially N-fixing clade. See Fig. 4 caption and Methods for explanation of *ST_W_*. Vertical bars are arithmetic means.

The phylogeny of FIA angiosperm genera (Fig. 3) shows that *ST_W_* has large ranges both within and outside the potentially N-fixing clade. (Figs. S4, S5, S6 and S7 show the phylogeny with the other successional indices.) Among N-fixing tree taxa in the U.S., *ST_W_* had lower maxima and means for N-fixing than non-fixing tree taxa in the U.S., when measured at either the genus or species level (Fig. 4; see Fig. S8 for *ST_U_*). Thus, both species- and genus-level *ST_W_* distributions indicate that N fixers are less shade tolerant than non-fixers within and outside the potentially N-fixing clade (Fig. 4). In contrast, non-fixers within and outside of the potentially N-fixing clade have similar distributions of *ST_W_* (Fig. 4C–F). As shown in Table 1, PICs indicate that the negative evolutionary relationships between N fixation and shade tolerance are significant (PICs with *ST_U_*, not shown, reveal nearly identical significance to *ST_W_*).

**Table 1:** Results from genus-level analyses of phylogenetically independent contrasts of N fixation versus successional indices.

Successional index*	Phylogenetic scale†	N fixation‡	df	*t* value	*P* value¶
*ST_W_*	Angiosperm	discrete	3	−3.79	**0.016**
*ST_W_*	Angiosperm	continuous	48	−4.21	**<0.001**
*ST_W_*	PNFC	discrete	3	−3.79	**0.016**
*ST_W_*	PNFC	continuous	25	−3.87	**<0.001**
*SA_W-mean_*	Angiosperm	discrete	3	−0.93	0.211
*SA_W-mean_*	Angiosperm	continuous	48	−3.58	**<0.001**
*SA_W-mean_*	PNFC	discrete	3	−0.93	0.211
*SA_W-mean_*	PNFC	continuous	25	−2.89	**0.004**
*SA_W-max_*	Angiosperm	discrete	3	−2.28	**0.053**
*SA_W-max_*	Angiosperm	continuous	48	−3.39	**<0.001**
*SA_W-max_*	PNFC	discrete	3	−2.28	**0.053**
*SA_W-max_*	PNFC	continuous	25	−3.00	**0.003**

*ST_W_: shade tolerance, geographically weighted by shade tolerance in each grid cell; *SA_W-mean_* and *SA_W-max_*: mean stand age, geographically weighted by mean and maximum stand age in each grid cell, respectively.

† Each test was performed at two phylogenetic scales: all U.S. angiosperm trees and the potentially N-fixing clade (PNFC; a subclade of the angiosperm phylogeny).

‡ ‘Discrete’ means that each genus was treated as an N fixer or not, whereas ‘continuous’ means that each genus was assigned the proportion of species that fix N (this proportion is either 0 or 1 in all U.S. tree genera).

¶ *P* values are from one tailed tests. Significant or marginally significant *P* values are in bold.

### Stand age and N fixation

**Figure 5:**
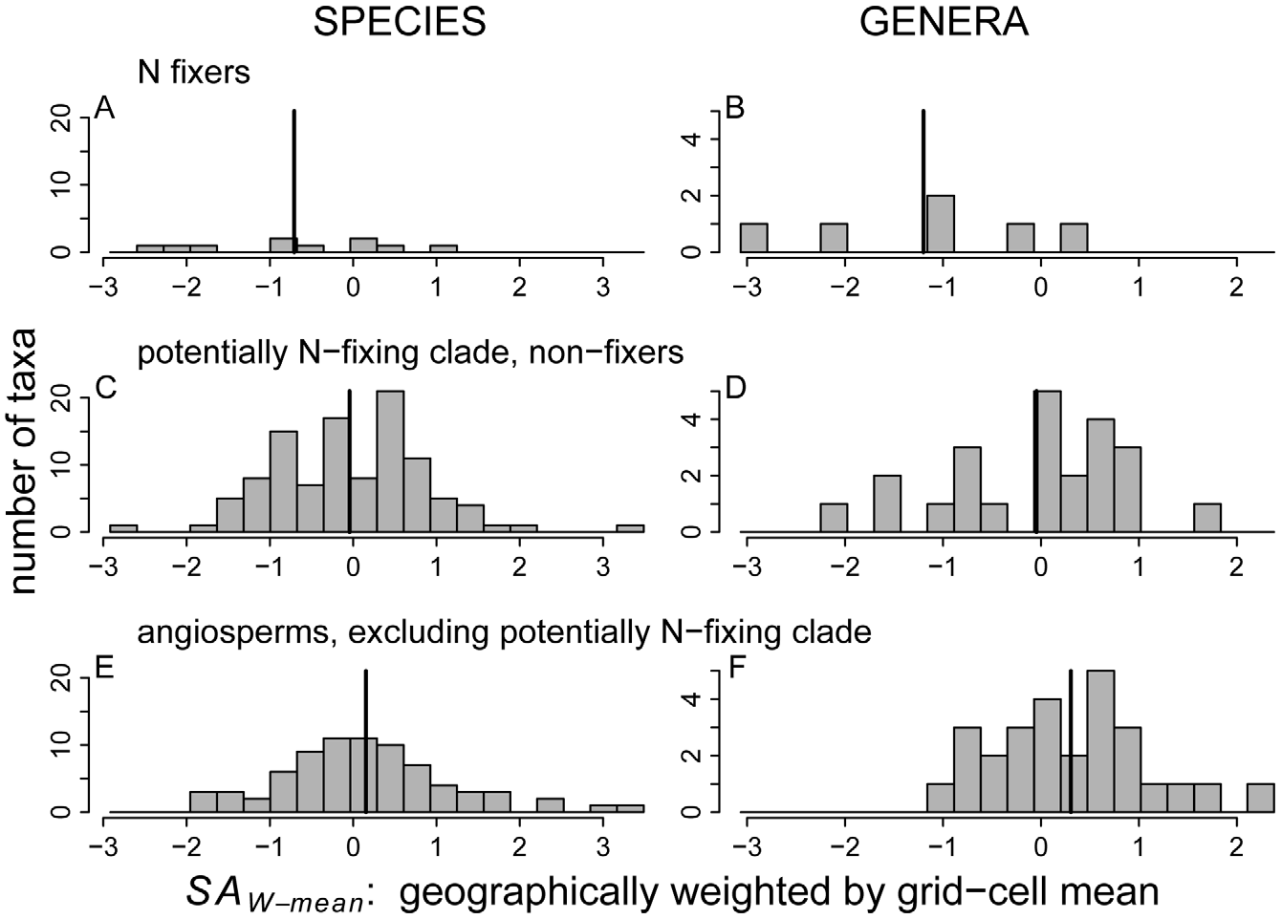
Histograms of the geographically weighted stand age index, *SA_W-mean_*. Panels are defined as in Fig. 4. *SA_W-mean_* accounts for geographical variation in mean stand age (Fig. S3), and is expressed as the number of standard deviations from the overall angiosperm mean (see Methods for details).

**Figure 6:**
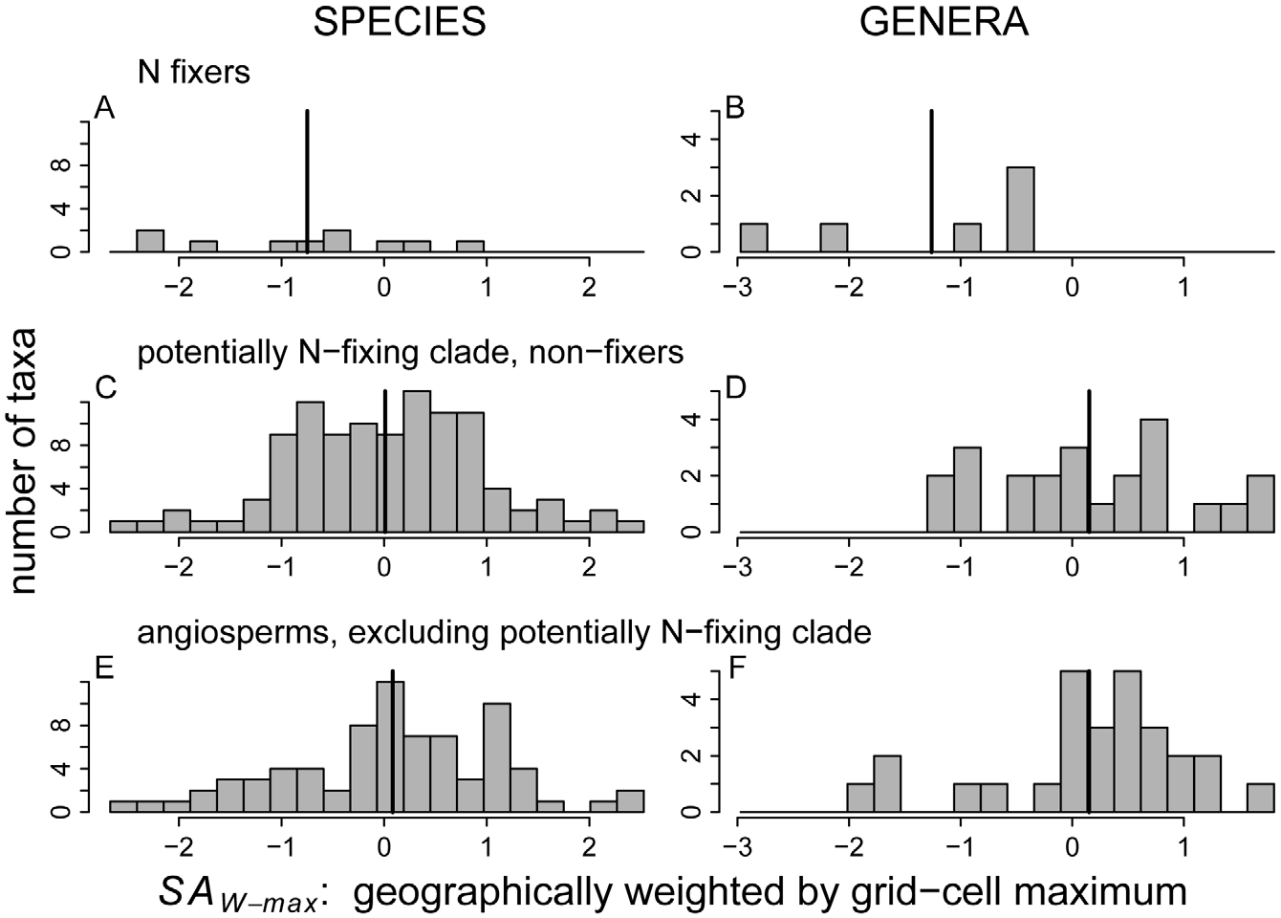
Histograms of the geographically weighted stand age index, *SA_W-max_*. Panels are defined as in Fig. 4. *SA_W-max_* accounts for geographical variation in maximum stand age (Fig. S3), and is expressed as the number of standard deviations from the overall angiosperm mean (see Methods for details).

The geographically weighted indices of mean stand age in which taxa occurred (*SA_W-mean_* and *SA_W-max_*) show that N fixers (Fig. 5A,B; 6A,B) tend to occur in relatively young stands within a given region (Fig. 5C–F, 6C–F; see Fig. S9 for *SA_U_*). These negative evolutionary relationships between N fixation and geographically weighted *SA* are significant for the continuous treatment of N fixation but marginally (*SA_W-max_*) or not (*SA_W-mean_*) significant for the discrete (more conservative) treatment (Table 1).

### Phylogenetic signal

The N fixation trait, whether treated as discrete or continuous and regardless of which phylogeny was used, was significantly conserved (*P*<0.05). There was no evidence for phylogenetic signal of weighted shade tolerance (*P*>0.10 for *ST_W_*) or weighted stand age (*P*>0.20 for *SA_W-mean_* and *SA_W-max_*), indicating that they are not evolutionarily conserved.

## Discussion

We have shown that among tree taxa in the coterminous U.S., N fixers are less shade-tolerant than non-fixers. Accounting for the fact that most N-fixing U.S. tree taxa occur in the western U.S.—where forests tend to be older than in the eastern U.S.—reveals that N fixers also tend to occur in relatively young stands, as expected from their shade intolerance. The above results hold whether N fixers are compared to all non-fixing angiosperms or only candidate N fixers (non-fixers in the potentially N-fixing clade), although some comparisons are only marginally significant. The effect sizes are large and consistent, so the lack of universally strong statistical support for these patterns likely reflects the small number of N-fixing tree genera available for analysis (six) and the close relatedness of some of these taxa.

Our findings that N fixers tend to be shade intolerant and occur in relatively young forests are consistent with the widely held but previously untested view that N fixers are primarily early successional in temperate biomes [1], [2], [4], [5]. Furthermore, our results suggest that this pattern cannot be explained by the phylogenetic constraints hypothesis, which posits that no temperate tree taxa are genetically predisposed to both fix N and be late successional. On the contrary, a number of shade-tolerant, late-successional U.S. tree taxa, such as *Fagus* (beech) and *Carya* (hickory), are in the potentially N-fixing clade, and thus are candidate N fixers. Overall, more than half of angiosperm tree species and genera in the U.S. are in the potentially N-fixing clade, indicating an abundance of candidate N fixers. Given this abundance of candidate N fixers, the negative evolutionary relationship between N fixation and successional status suggests that late-successional N fixers have been selected against. Therefore, it suggests that the rarity of late-successional, temperate N-fixing trees is due to selective (e.g., ecological or physiological), rather than phylogenetic, constraints.

Although our analyses are restricted to U.S. tree taxa, anecdotal evidence suggests that the association of N fixation with an early-successional, shade-intolerant life history may hold within temperate zones more broadly. For instance, N-fixing shrubs in the U.S. are primarily found in pioneer stages of forests or other open habitats, supporting the relationship between shade intolerance and N fixation. The actinorhizal genus *Ceanothus* is common in open, recently disturbed forests of the Pacific Northwest [10], [46], [47] and Mountain West [48], and also in fire-prone California chaparral [10], [11], [12]. Other actinorhizal genera in the U.S. are also primarily found in open areas—such as *Shepherdia canadensis*, *Cercocarpus montanus*, and *Elaeagnus commutata* [47], [48]—and some, such as *Dryas drummondii*, are pioneer species that tend to occur prior to tree-dominated stages of succession [47]. Invasive leguminous shrubs, such as *Cytisus scoparius* and *Ulex europaeus*, and the majority of herbaceous legumes are also found in open, disturbed areas [47], [48]. Temperate habitats in New Zealand follow a similar pattern: The actinorhizal *Coriaria arborea*, *C. pteridoides*, and *C. plumose* are common pioneer shrubs and/or trees in many parts of the country, and are rarely if ever found in late-successional forests [49], [50], [51]. Similarly, the native leguminous shrubs *Carmichaelia grandifolia* and *C. odorata* are found primarily early in succession [51], [52]. An exception to the pattern may be temperate forests of southern Australia and Tasmania, where *Acacia melanoxylon* is commonly found in the subcanopy as well as the canopy [53].

Current evidence favors multiple origins for the evolution of N fixation (see Phylogenetic context section) rather than a single origin and multiple subsequent losses [26], but our conclusion that phylogenetic constraints cannot explain the rarity of late-successional N fixers is consistent with either possibility. (i) If N fixation evolved only once (at the base of the potentially N-fixing clade), the trait was preferentially lost from late-successional taxa as the original N-fixing ancestor diversified to cover the full range of successional strategies observed among angiosperms (Figs. 3, S4, S5, S6 and S7). (ii) If N fixation evolved multiple times, it evolved preferentially in early-successional taxa. Assuming that mutations conferring the ability to fix N have appeared without bias across the potentially N-fixing clade, N fixation has likely appeared in late-successional taxa, but the combination of N fixation and a late-successional strategy has been selected against. Moreover, the lack of significant conservatism of the successional traits suggests that a lineage could evolve the capacity to be late successional following the appearance of N fixation if this combination of traits were not at a selective disadvantage. The above considerations all support the conclusion that the rarity of N fixers in late-successional temperate forests likely results from selective rather than phylogenetic constraints.

Selection against the combination of N fixation and late-successional traits implies that there are ecological or physiological tradeoffs with N fixation that affect some traits that are ecologically related to successional status. A number of studies have suggested or documented potential tradeoffs between N fixation and other traits. For example, it is well known that the energetic cost of N fixation is high relative to other forms of N acquisition [54], at least when other forms of N are abundantly available. Therefore, when energetic constraints are high (e.g., when plants are shaded), N fixation might not be adaptive [16]. However, given that many N fixers are facultative [6], [55], [56]—i.e., they can down-regulate N fixation—energetic constraints alone do not obviously explain why N fixation would be selected against in canopy-level (i.e., unshaded) late-successional trees. In addition to energetic constraints, N fixers may have lower N use efficiency [17], higher susceptibility to herbivores [2], [17], [19], higher demand for another resource [2], [16], [19], and/or lower uptake of other N forms such as nitrate or ammonium [16], [17] compared to non-fixers. The ends of these spectra associated with N fixation—low N use efficiency, poor herbivore defense, high requirements for other resources, and a poor ability to deplete soil nutrients—are typically associated with shade-intolerant, early-successional tree taxa. There is evidence for some of these tradeoffs [e.g., 18], and rough calculations suggest that they may be strong enough to select against N fixers in late-successional forests, even those that are purely N-limited [17]. The case is far from closed, though, and future studies targeting these tradeoffs would be welcome.

In contrast to the pattern in temperate forests, tropical forests are rife with canopy trees that are capable of N fixation [5]. It is generally thought that some of these are late successional [5], [9], but to our knowledge the shade tolerance and successional status of tropical N fixers has not been quantified at broad geographic and taxonomic scales, as we have done here for N-fixing trees in the coterminous U.S. The prevalence of N fixation among shade-tolerant, late-successional tropical trees, if confirmed, would extend to a broader geographical area our conclusion that there are no phylogenetic constraints to late-successional N-fixation. Furthermore, the prevalence of late-successional tropical N fixers would necessitate an explanation for why selective constraints differ between temperate and tropical biomes.

Although atmospheric N deposition is currently high in some temperate forests relative to most of the tropics [57], and this has induced N saturation in parts of the eastern U.S. [58], [59] and northern Europe [60], it is unlikely to explain the biome-level difference in N-fixing tree abundance or the rarity of late-successional temperate N-fixing trees for three primary reasons. First, many tropical forests seem to be naturally N rich even without large atmospheric N deposition inputs [9], so they are likely as N saturated as polluted temperate forests. Second, many temperate areas such as the western U.S., Chile, and New Zealand have low atmospheric N deposition [57] yet still generally lack late-successional N fixers. Third, the current late-successional community structure is largely determined by pre-industrial environmental conditions, when atmospheric N deposition was much lower worldwide (<5 kg N ha^−1^ y^−1^) than current rates in polluted areas (>20 kg N ha^−1^ yr^−1^) [57].

What else could explain an abundance of late-successional tropical N-fixing trees? Temperature and growing season length are obvious differences between the two biomes, and have been suggested to play roles in the pattern [20], [61]. The nitrogenase enzyme has a temperature optimum around 25°C, which may make N fixation a more profitable strategy in tropical biomes [61], although its temperature response relative to other N processing enzymes remains unexplored. Many tropical leguminous N-fixing trees seem to down-regulate N fixation when soil N availability is high [55], [56], whereas many temperate actinorhizal N-fixing trees do not [1], [62]. This difference in N fixation strategy can explain the biome-level difference in N fixer success [9], [20], and could result from different growing season lengths or temperatures [20], but this question remains open.

N fixation could play a critical role in mitigating anthropogenic CO_2_ emissions [15] by stimulating increased primary production in N-limited areas, thereby removing additional CO_2_ from the atmosphere. For example, primary production is more responsive to experimental CO_2_ enrichment when N availability is also increased experimentally [63]. Temperate forests are often N-limited [2], [13], [64], which may contribute to the absence of a CO_2_ fertilization signal in eastern U.S. forest inventory data [65]. These observations highlight the need to understand the factors controlling the distributions of N-fixing trees. Our results from the coterminous U.S. support the notion that N fixers are confined primarily to an early-successional role in temperate forests, but suggest that this confinement does not stem from phylogenetic constraints; rather, it likely results from selective tradeoffs between N fixation and other traits. Understanding these tradeoffs would be an important step towards understanding the broad-scale controls on the N and carbon cycles.

## Supporting Information

Figure S1 Geographical patterns of N fixer basal area by species. Values are the species' percentage of total basal area in the grid cell. See text and Fig. 2 caption for details. Note the different scale in each panel.
(0.32 MB PDF)

Figure S2 Relationship between the unweighted and weighted shade tolerance indices and a well-known categorical classification. The categorical classification is from *Silvics of North America* [27]. (A) *ST_U_* is the raw proportion of saplings of each species in the FIA data with crown class ‘overtopped’ or ‘intermediate,’ and (B) *ST_W_* is *ST_U_* geographically-weighted relative to the mean value in 2°×2° grid cells (see Methods for details). All 156 species (including conifers) that are classified in Silvics and with at least 20 live FIA saplings with a reported crown class are included. The figure displays standard box-plots: Bold bars are medians, boxes indicate the first and third quartiles, error bars are the most extreme points within 1.5 interquartile ranges of the first and third quartiles, and circles are outliers (all points outside of the error bars).
(0.01 MB PDF)

Figure S3 Geographical patterns of successional indices. (A) Mean stand age (years), (B) maximum stand age (years), and (C) proportion of saplings (all taxa combined) in the understory (‘overtopped’ or ‘intermediate’ FIA crown class). See text and Fig. 2 caption for details. White spaces reflect grid cells in which fewer than 20 values (i.e., plots with a reported stand age, or saplings with a reported crown class) were available.
(0.22 MB PDF)

Figure S4 Character history reconstruction of the geographically unweighted shade tolerance index (*ST_U_*) for angiosperm FIA genera. See text and Fig. 3 caption for details.
(0.38 MB PDF)

Figure S5 Character history reconstruction of the geographically unweighted stand age index (*SA_U_*) for angiosperm FIA genera. See text and Fig. 3 caption for details.
(0.38 MB PDF)

Figure S6 Character history reconstruction of the geographically weighted stand age index (*SA_W-mean_*) for angiosperm FIA genera. See text and Fig. 3 caption for details.
(0.38 MB PDF)

Figure S7 Character history reconstruction of the geographically weighted stand age index (*SA_W-max_*) for angiosperm FIA genera. See text and Fig. 3 caption for details.
(0.38 MB PDF)

Figure S8 Histograms of the geographically unweighted shade tolerance index (*ST_U_*). Panels are defined as in Fig. 4. *ST_U_* (unitless) is the proportion of live saplings in the FIA data with an understory (as opposed to canopy) crown class (see Methods for details).
(0.17 MB PDF)

Figure S9 Histograms of the geographically unweighted stand age index (*SA_U_*). Panels are defined as in Fig. 4. *SA_U_* is the mean age of forest stands (years) in which each taxon occurs (see Methods for details).
(0.17 MB PDF)

Table S1 Percent of regional N fixer basal comprised by each N-fixing species, calculated from FIA data.
(0.05 MB DOC)

Table S2 Summary of FIA angiosperm data.
(0.45 MB DOC)
