# Supplementary material for: Phylogenetic Constraints Do Not Explain the Rarity of Nitrogen-Fixing Trees in Late-Successional Temperate Forests
Source: PLoS One. 2010 Aug 6;5(8):e12056. doi: 10.1371/journal.pone.0012056 (PMC2917374; doi:10.1371/journal.pone.0012056)
Supplement: Figure S1 — Geographical patterns of N fixer basal area by species. Values are the species' percentage of total basal area in the grid cell. See text and Fig. 2 caption for details. Note the different scale in each panel. (0.32 MB PDF) [file pone.0012056.s001.pdf]

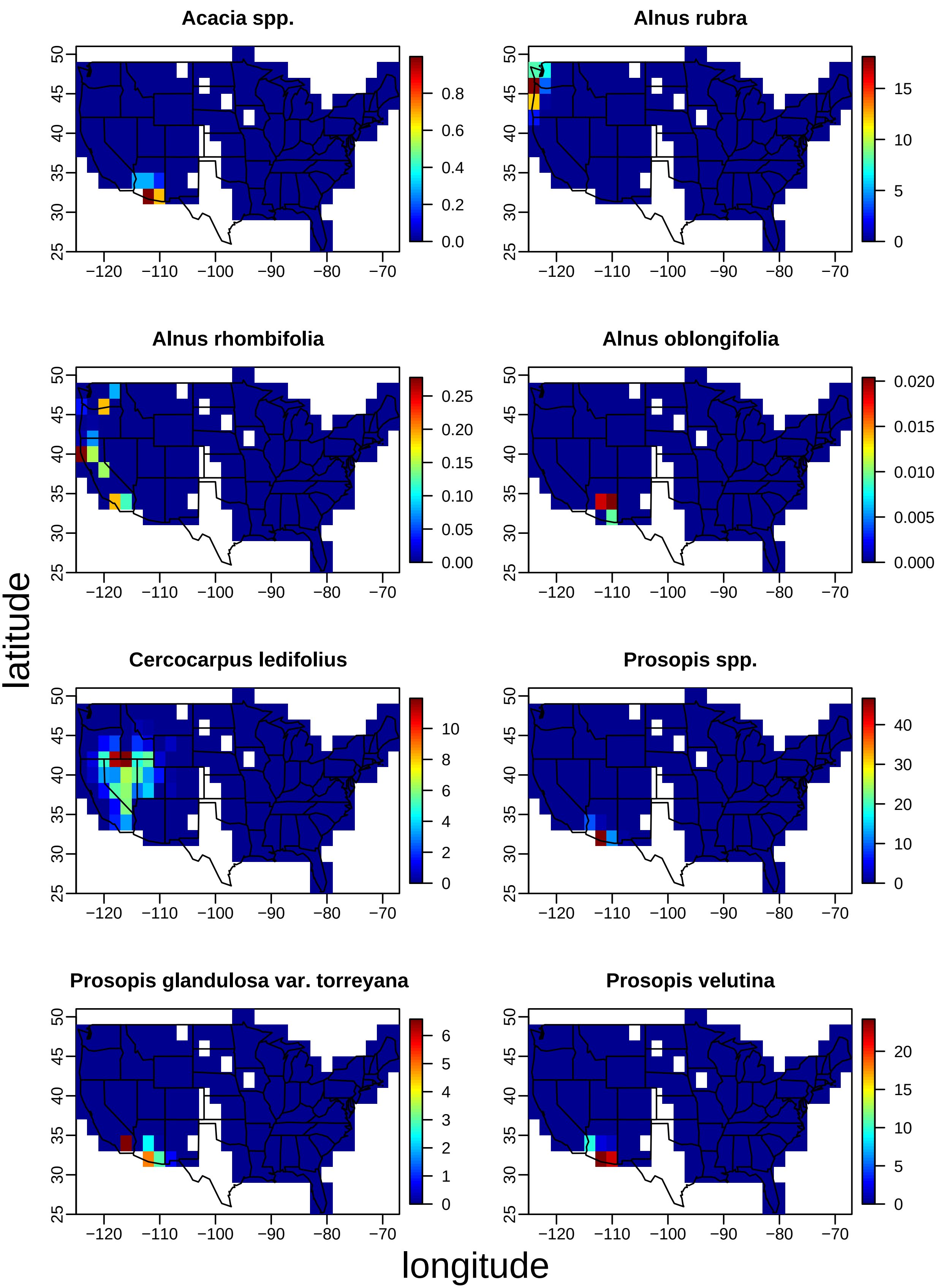
Acacia spp.
Alnus rubra
Alnus rhombifolia
Alnus oblongifolia
Cercocarpus ledifolius
Prosopis spp.
Prosopis glandulosa var. torreyana
Prosopis velutina
latitude
longitude
25 30 35 40 45 50
−120 −110 −100 −90 −80 −70
0.0 0.2 0.4 0.6 0.8
0 5 10 15
0.00 0.05 0.10 0.15 0.20 0.25
0.000 0.005 0.010 0.015 0.020
0 2 4 6 8 10
0 10 20 30 40
0 1 2 3 4 5 6
0 5 10 15 20

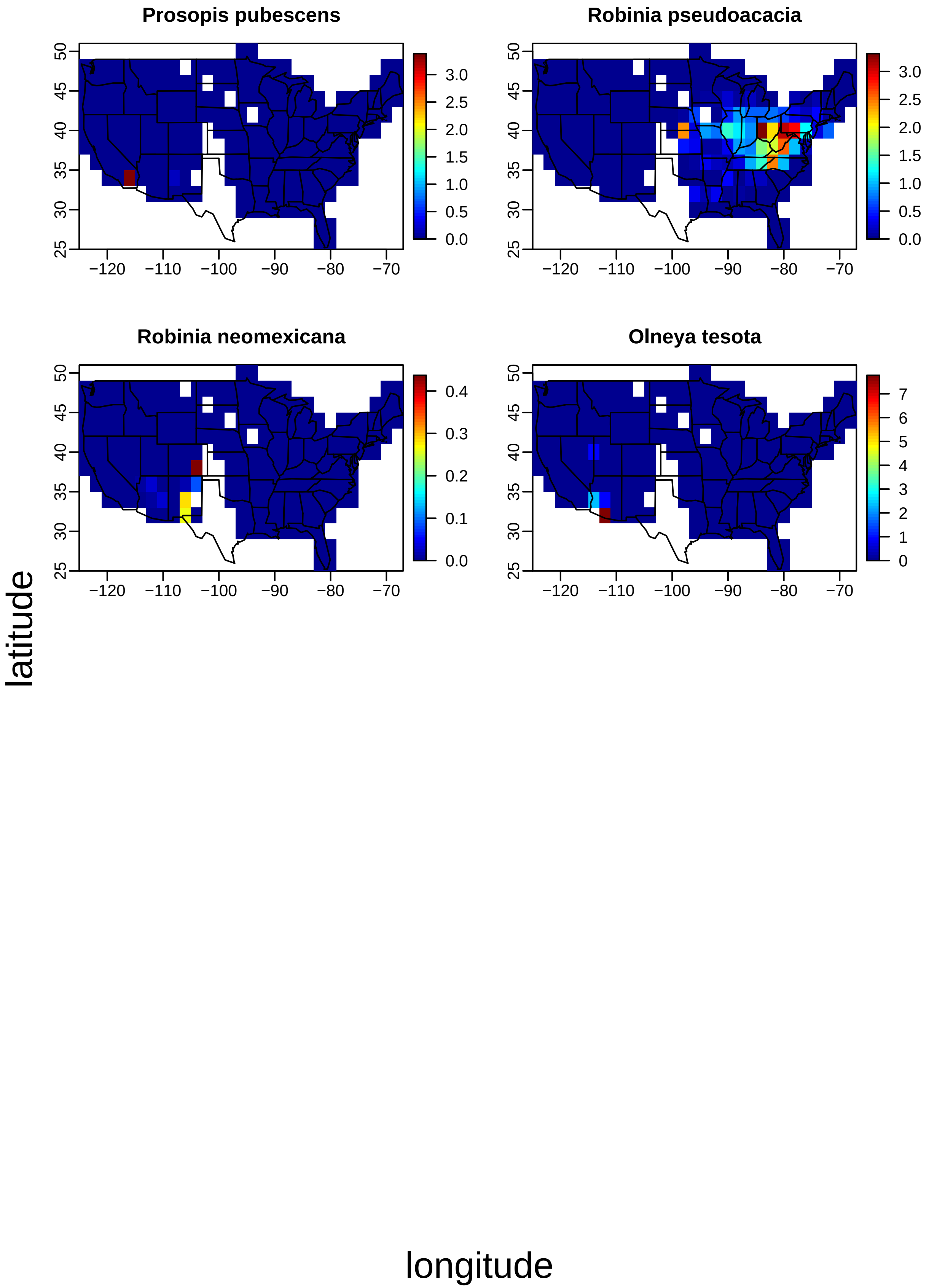
Prosopis pubescens
Robinia pseudoacacia
Robinia neomexicana
Olneya tesota
50
45
40
35
30
25
−120
−110
−100
−90
−80
−70
3.0
2.5
2.0
1.5
1.0
0.5
0.0
0.4
0.3
0.2
0.1
7
6
5
4
3
2
1
0
latitude
longitude
