# Supplementary figures and images for: Phylogenetic Constraints Do Not Explain the Rarity of Nitrogen-Fixing Trees in Late-Successional Temperate Forests
Source: PLoS One. 2010 Aug 6;5(8):e12056. doi: 10.1371/journal.pone.0012056 (PMC2917374; doi:10.1371/journal.pone.0012056)

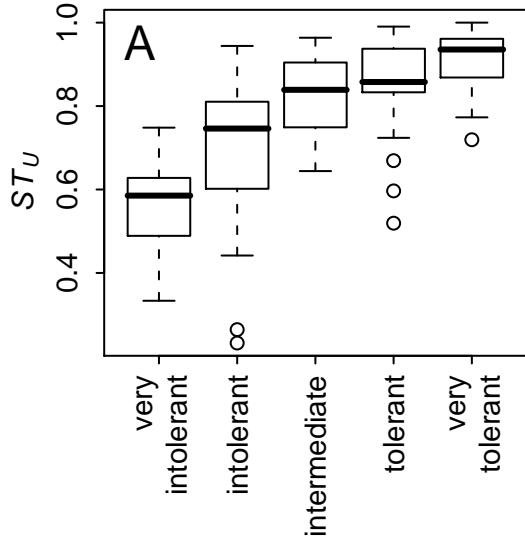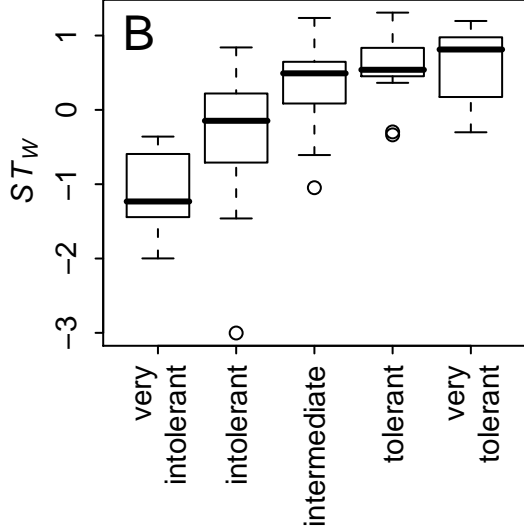

Supplement: Figure S2 — Relationship between the unweighted and weighted shade tolerance indices and a well-known categorical classification. The categorical classification is from Silvics of North America [27]. (A) STU is the raw proportion of saplings of each species in the FIA data with crown class ‘overtopped’ or ‘intermediate,’ and (B) STW is STU geographically-weighted relative to the mean value in 2°×2° grid cells (see Methods for details). All 156 species (including conifers) that are classified in Silvics and with at least 20 live FIA saplings with a reported crown class are included. The figure displays standard box-plots: Bold bars are medians, boxes indicate the first and third quartiles, error bars are the most extreme points within 1.5 interquartile ranges of the first and third quartiles, and circles are outliers (all points outside of the error bars). (0.01 MB PDF) [file pone.0012056.s002.pdf]

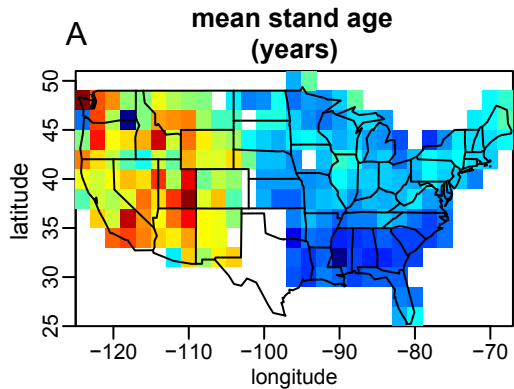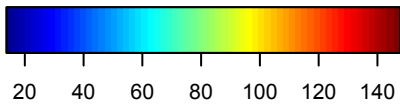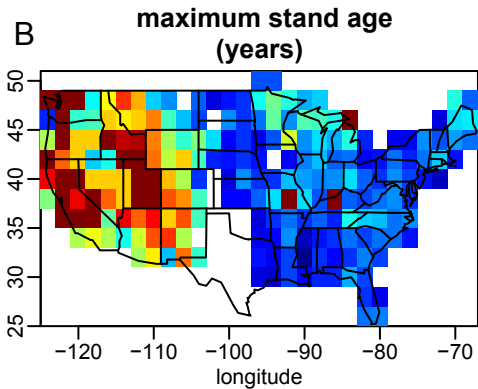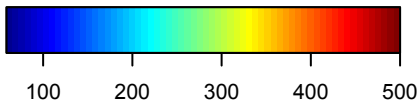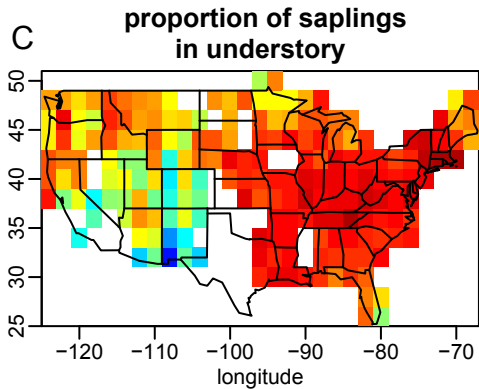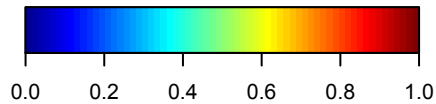

Supplement: Figure S3 — Geographical patterns of successional indices. (A) Mean stand age (years), (B) maximum stand age (years), and (C) proportion of saplings (all taxa combined) in the understory (‘overtopped’ or ‘intermediate’ FIA crown class). See text and Fig. 2 caption for details. White spaces reflect grid cells in which fewer than 20 values (i.e., plots with a reported stand age, or saplings with a reported crown class) were available. (0.22 MB PDF) [file pone.0012056.s003.pdf]

$ST_U$  (unitless)

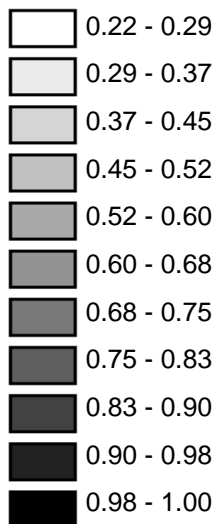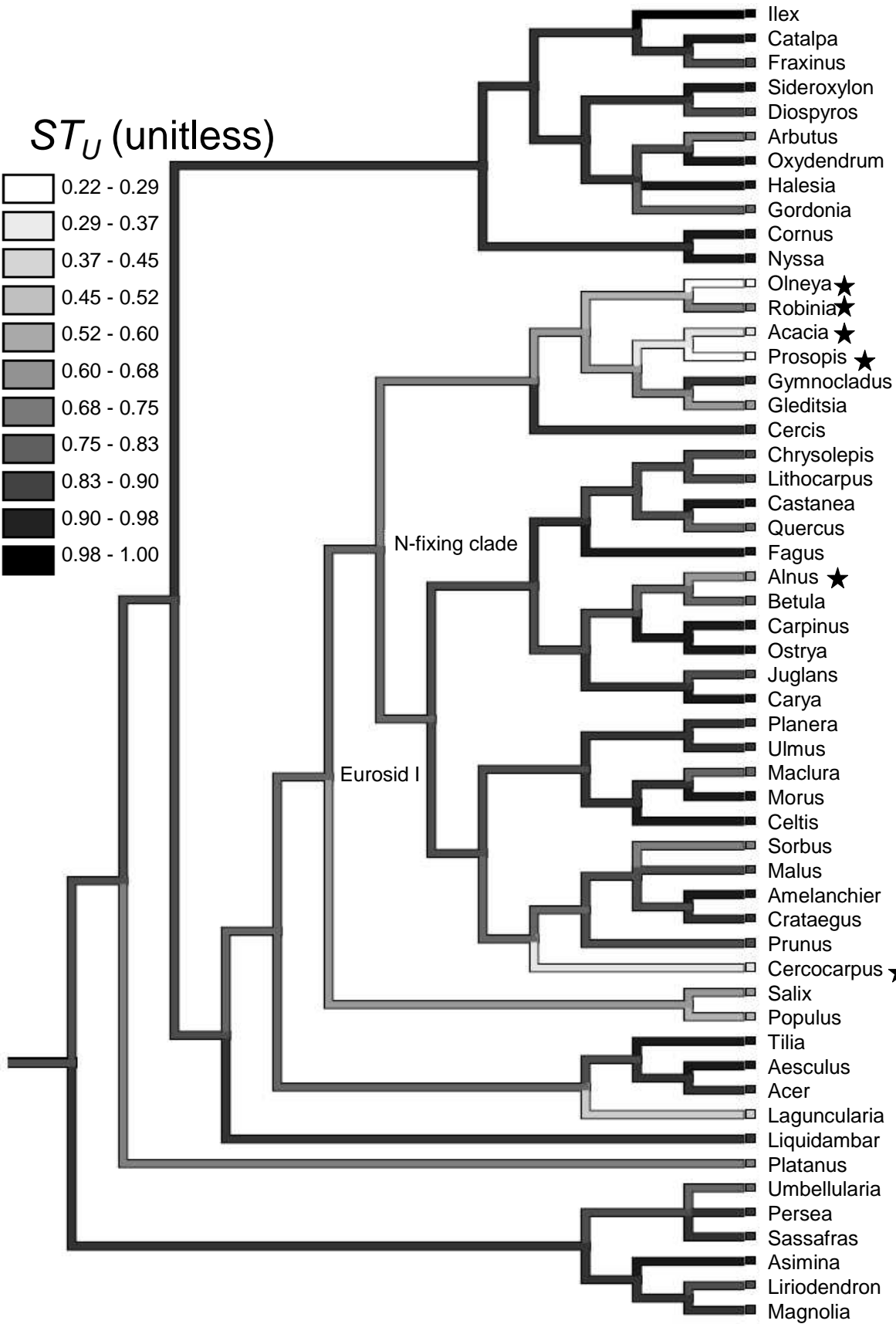

Supplement: Figure S4 — Character history reconstruction of the geographically unweighted shade tolerance index (STU) for angiosperm FIA genera. See text and Fig. 3 caption for details. (0.38 MB PDF) [file pone.0012056.s004.pdf]

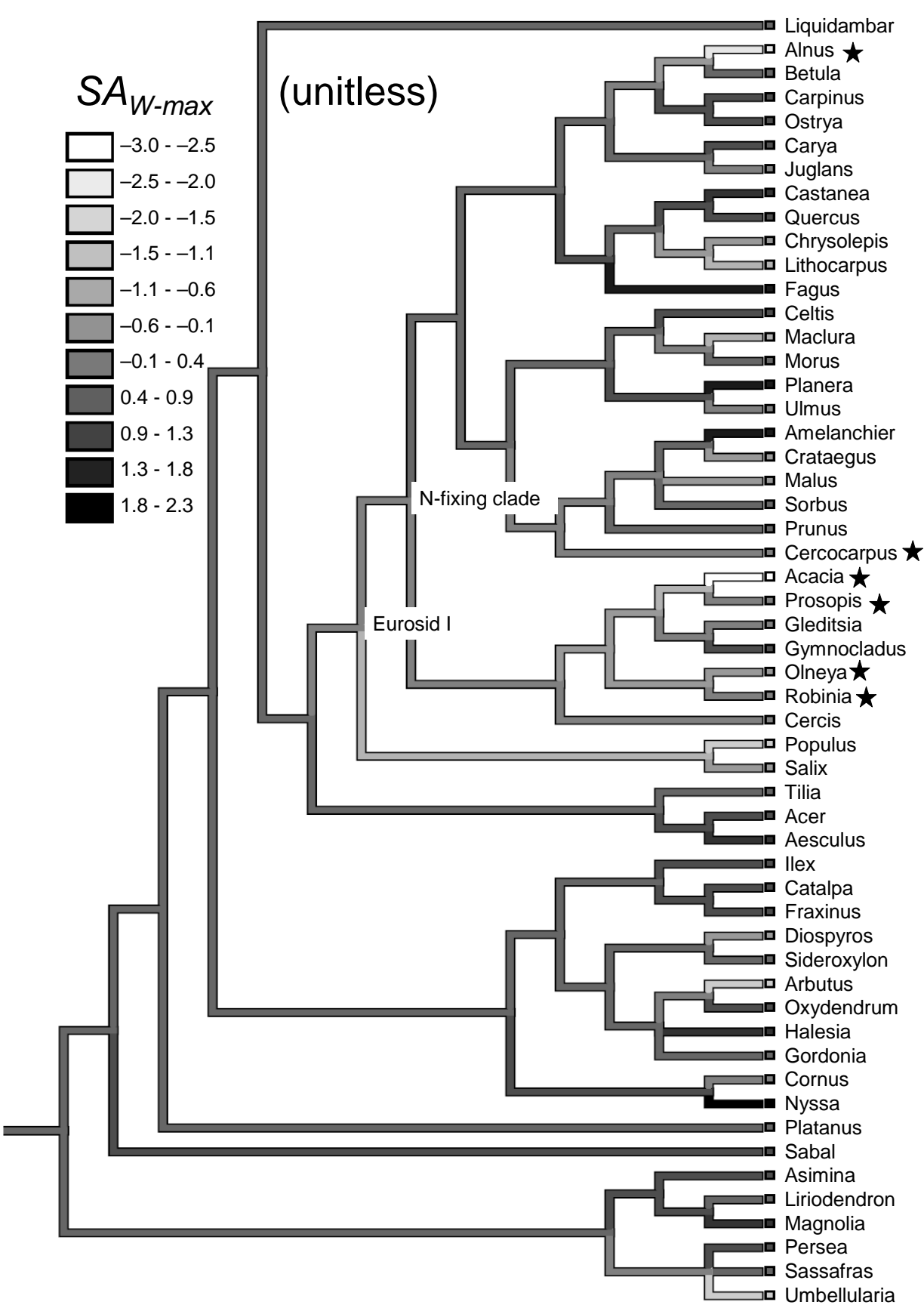

Supplement: Figure S7 — Character history reconstruction of the geographically weighted stand age index (SAW-max) for angiosperm FIA genera. See text and Fig. 3 caption for details. (0.38 MB PDF) [file pone.0012056.s007.pdf]
