# Supplementary material for: Phylogenetic Constraints Do Not Explain the Rarity of Nitrogen-Fixing Trees in Late-Successional Temperate Forests
Source: PLoS One. 2010 Aug 6;5(8):e12056. doi: 10.1371/journal.pone.0012056 (PMC2917374; doi:10.1371/journal.pone.0012056)
Supplement: Figure S5 — Character history reconstruction of the geographically unweighted stand age index (SAU) for angiosperm FIA genera. See text and Fig. 3 caption for details. (0.38 MB PDF) [file pone.0012056.s005.pdf]

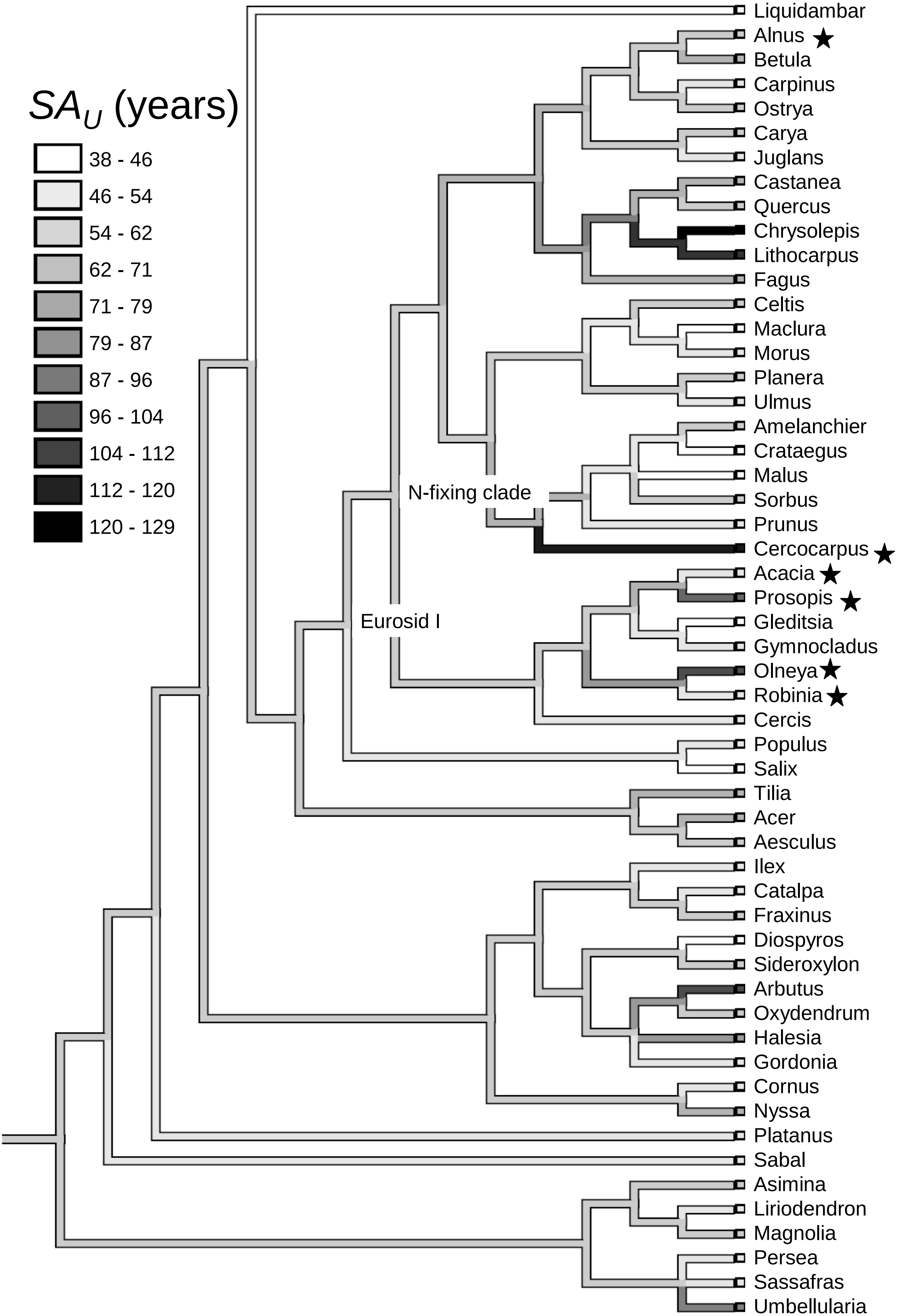

SA_U (years)
38 - 46
46 - 54
54 - 62
62 - 71
71 - 79
79 - 87
87 - 96
96 - 104
104 - 112
112 - 120
120 - 129
N-fixing clade
Eurosid I
Liquidambar
Alnus ★
Betula
Carpinus
Ostrya
Carya
Juglans
Castanea
Quercus
Chrysolepis
Lithocarpus
Fagus
Celtis
Maclura
Morus
Planera
Ulmus
Amelanchier
Crataegus
Malus
Sorbus
Prunus
Cercocarpus ★
Acacia ★
Prosopis ★
Gleditsia
Gymnocladus
Olneya ★
Robinia ★
Cercis
Populus
Salix
Tilia
Acer
Aesculus
Ilex
Catalpa
Fraxinus
Diospyros
Sideroxylon
Arbutus
Oxydendrum
Halesia
Gordonia
Cornus
Nyssa
Platanus
Sabal
Asimina
Liriodendron
Magnolia
Persea
Sassafras
Umbellularia
