# Supplementary material for: Phylogenetic Constraints Do Not Explain the Rarity of Nitrogen-Fixing Trees in Late-Successional Temperate Forests
Source: PLoS One. 2010 Aug 6;5(8):e12056. doi: 10.1371/journal.pone.0012056 (PMC2917374; doi:10.1371/journal.pone.0012056)
Supplement: Figure S6 — Character history reconstruction of the geographically weighted stand age index (SAW-mean) for angiosperm FIA genera. See text and Fig. 3 caption for details. (0.38 MB PDF) [file pone.0012056.s006.pdf]

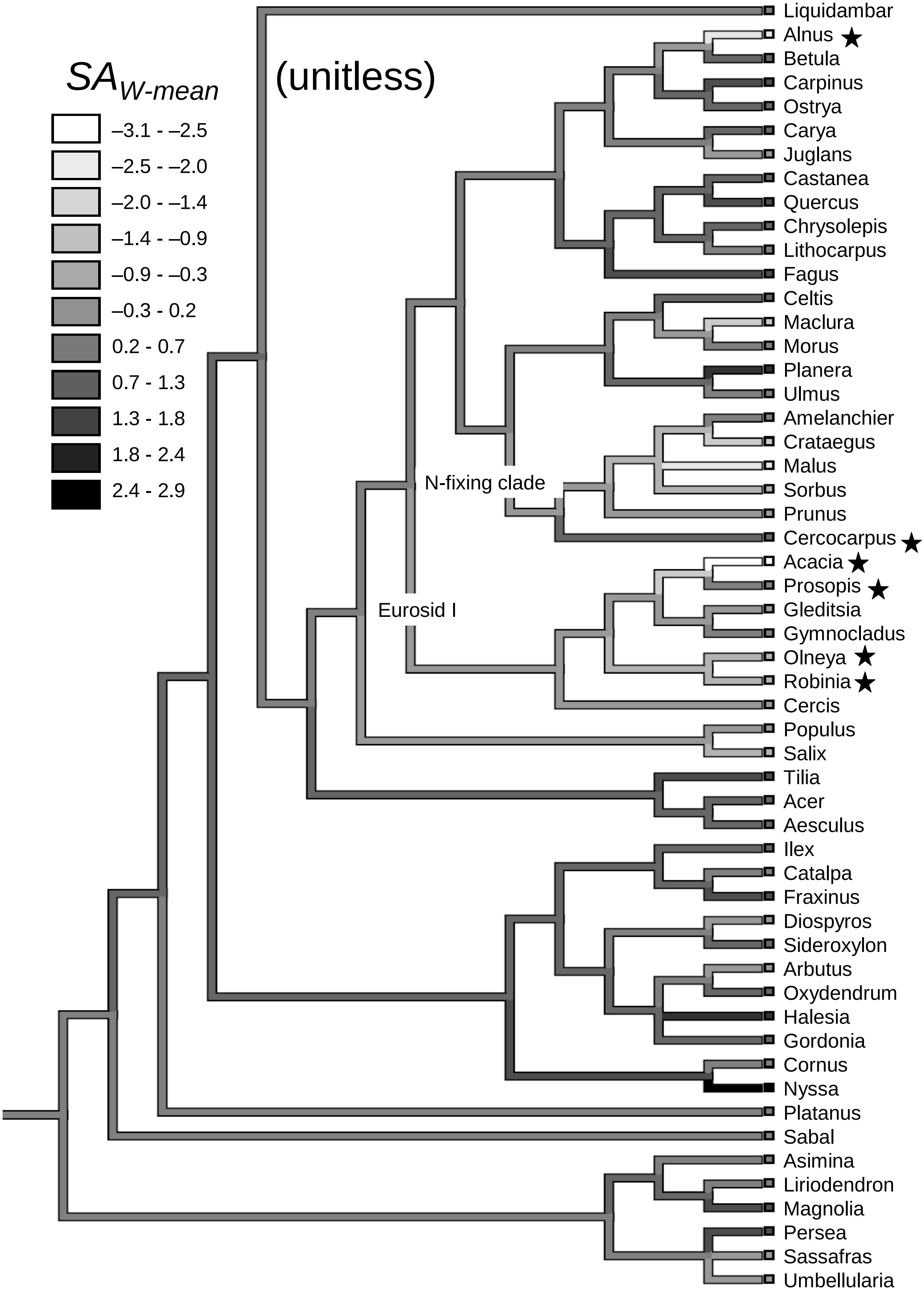

$SA_{W\text{-}mean}$
(unitless)
–3.1 - –2.5
–2.5 - –2.0
–2.0 - –1.4
–1.4 - –0.9
–0.9 - –0.3
–0.3 - 0.2
0.2 - 0.7
0.7 - 1.3
1.3 - 1.8
1.8 - 2.4
2.4 - 2.9
N-fixing clade
Eurosid I
Liquidambar
Alnus ★
Betula
Carpinus
Ostrya
Carya
Juglans
Castanea
Quercus
Chrysolepis
Lithocarpus
Fagus
Celtis
Maclura
Morus
Planera
Ulmus
Amelanchier
Crataegus
Malus
Sorbus
Prunus
Cercocarpus ★
Acacia ★
Prosopis ★
Gleditsia
Gymnocladus
Olneya ★
Robinia ★
Cercis
Populus
Salix
Tilia
Acer
Aesculus
Ilex
Catalpa
Fraxinus
Diospyros
Sideroxylon
Arbutus
Oxydendrum
Halesia
Gordonia
Cornus
Nyssa
Platanus
Sabal
Asimina
Liriodendron
Magnolia
Persea
Sassafras
Umbellularia
