# Supplementary material for: Phylogenetic Constraints Do Not Explain the Rarity of Nitrogen-Fixing Trees in Late-Successional Temperate Forests
Source: PLoS One. 2010 Aug 6;5(8):e12056. doi: 10.1371/journal.pone.0012056 (PMC2917374; doi:10.1371/journal.pone.0012056)
Supplement: Figure S8 — Histograms of the geographically unweighted shade tolerance index (STU). Panels are defined as in Fig. 4. STU (unitless) is the proportion of live saplings in the FIA data with an understory (as opposed to canopy) crown class (see Methods for details). (0.17 MB PDF) [file pone.0012056.s008.pdf]

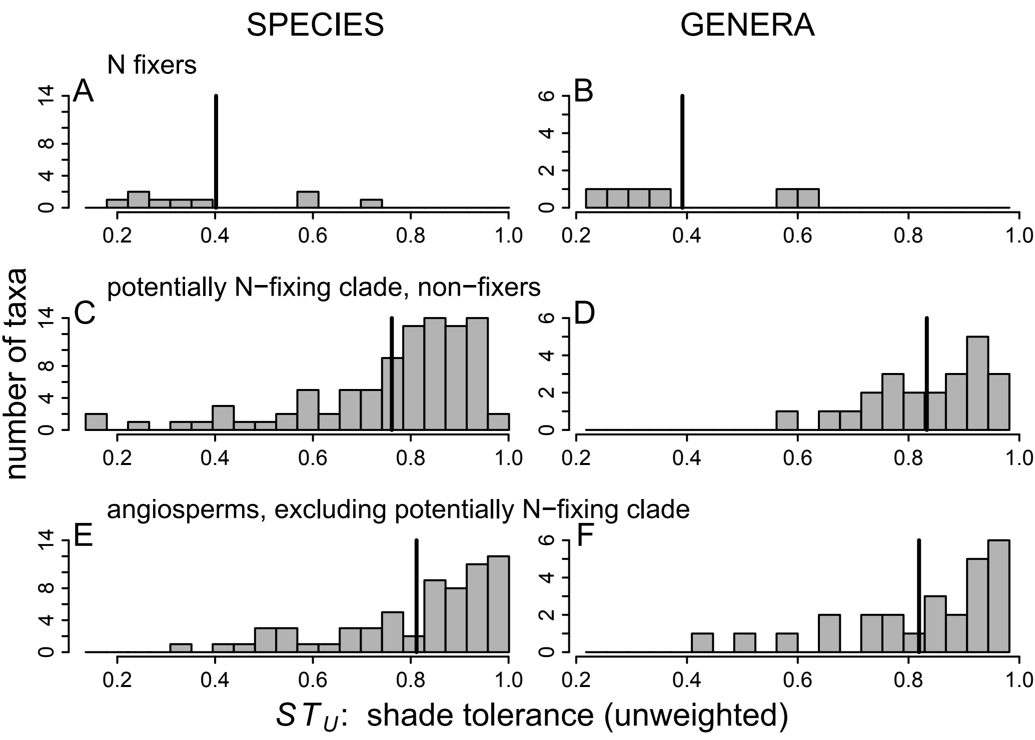

SPECIES
GENERA
N fixers
A
B
potentially N−fixing clade, non−fixers
C
D
angiosperms, excluding potentially N−fixing clade
E
F
number of taxa
0
4
8
14
0
2
4
6
0.2
0.4
0.6
0.8
1.0
ST_U: shade tolerance (unweighted)
