# Supplementary material for: Phylogenetic Constraints Do Not Explain the Rarity of Nitrogen-Fixing Trees in Late-Successional Temperate Forests
Source: PLoS One. 2010 Aug 6;5(8):e12056. doi: 10.1371/journal.pone.0012056 (PMC2917374; doi:10.1371/journal.pone.0012056)
Supplement: Figure S9 — Histograms of the geographically unweighted stand age index (SAU). Panels are defined as in Fig. 4. SAU is the mean age of forest stands (years) in which each taxon occurs (see Methods for details). (0.17 MB PDF) [file pone.0012056.s009.pdf]

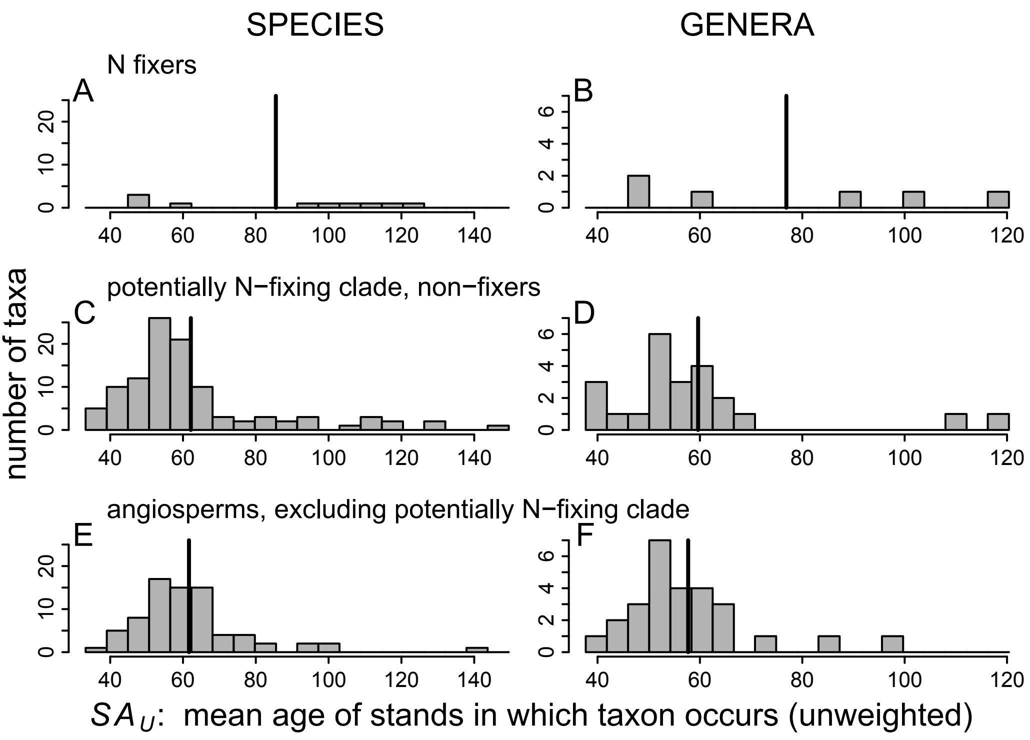

SPECIES
GENERA
N fixers
A
B
potentially N−fixing clade, non−fixers
C
D
angiosperms, excluding potentially N−fixing clade
E
F
number of taxa
0
10
20
0
2
4
6
40
60
80
100
120
140
SA_U: mean age of stands in which taxon occurs (unweighted)
