# Supplementary material for: Phylogenetic Constraints Do Not Explain the Rarity of Nitrogen-Fixing Trees in Late-Successional Temperate Forests
Source: PLoS One. 2010 Aug 6;5(8):e12056. doi: 10.1371/journal.pone.0012056 (PMC2917374; doi:10.1371/journal.pone.0012056)
Supplement: Table S1 — Percent of regional N fixer basal comprised by each N-fixing species, calculated from FIA data. (0.05 MB DOC) [file pone.0012056.s010.doc]

**Table S1**. Percent of regional N fixer basal comprised by each N-fixing species, calculated from FIA data

| **Genus** | **Species** | **EUS^*^** | **WUS^*^** | **WWAOR^*^** | **AZNM^*^** |
| --- | --- | --- | --- | --- | --- |
| *Acacia* | spp. |  | 0.38% |  | 2.22% |
| *Alnus* | *oblongifolia* |  | 0.17% |  | 1.01% |
| *Alnus* | *rhombifolia* |  | 0.77% | 0.10% |  |
| *Alnus* | *rubra* |  | 36.80% | 99.89% |  |
| *Cercocarpus* | *ledifolius* |  | 45.12% | 0.01% | <0.01% |
| *Olneya* | *tesota* |  | 0.52% |  | 2.31% |
| *Prosopis* | *glandulosa* var. *torreyana* |  | 1.74% |  | 10.11% |
| *Prosopis* | *pubescens* |  | 0.03% |  | 0.15% |
| *Prosopis* | spp. |  | 7.21% |  | 41.97% |
| *Prosopis* | *velutina* |  | 6.86% |  | 39.94% |
| *Robinia* | *neomexicana* |  | 0.39% |  | 2.30% |
| *Robinia* | *pseudoacacia* | 100.00% | <0.01% |  |  |
|  |  |  |  |  |  |
| % Basal area occupied by N fixers^†^ | | 0.482% | 1.323% | 4.322% | 1.401% |
| Mean total basal area of N fixers (m^2^ ha^-1^) | | 0.106 | 0.289 | 2.202 | 0.200 |

^*^ EUS: Eastern U. S. (east of 100º W longitude); WUS: Western U. S.; WWAOR: Western Washington and Oregon (all counties west of the crest of the Cascade Mountains); AZNM: Arizona and New Mexico.

^†^ Species present in the FIA documentation with zero basal area in current surveys are not included in this table. Some species are included here but not in Table A2, which only includes species represented by ≥ 20 individuals.
