# Supplementary material for: Phylogenetic Constraints Do Not Explain the Rarity of Nitrogen-Fixing Trees in Late-Successional Temperate Forests
Source: PLoS One. 2010 Aug 6;5(8):e12056. doi: 10.1371/journal.pone.0012056 (PMC2917374; doi:10.1371/journal.pone.0012056)
Supplement: Table S2 — Summary of FIA angiosperm data. (0.45 MB DOC) [file pone.0012056.s011.doc]

**Table S2**: Summary of FIA angiosperm data

| **Species** | **Family** | **N fixer^*^** | **# *ST^*^*** | ***ST_U_^*^*** | ***ST_W_^*^*** | **# *SA^*^*** | ***SA_U_^*^*** | ***SA_W-mean_^*^*** | ***SA_W-max_^*^*** |
| --- | --- | --- | --- | --- | --- | --- | --- | --- | --- |
| *Acacia* spp. | Fabaceae | 1 | 41 | 0.317 | -0.71 | 57 | 46 | -2.58 | -2.31 |
| *Alnus rhombifolia* | Betulaceae | 1 | 18 |  |  | 139 | 93 | -0.49 | -1.03 |
| *Alnus rubra* | Betulaceae | 1 | 113 | 0.602 | -0.90 | 2795 | 58 | -1.92 | -1.75 |
| *Cercocarpus ledifolius* | Rosaceae | 1 | 396 | 0.369 | -1.96 | 8059 | 118 | 0.07 | -0.42 |
| *Olneya tesota* | Fabaceae | 1 | 32 | 0.219 | -2.25 | 124 | 100 | -0.90 | -0.82 |
| *Prosopis glandulosa* var. *torreyana* | Fabaceae | 1 | 54 | 0.278 | -1.39 | 352 | 108 | 0.52 | 0.38 |
| *Prosopis* spp. | Fabaceae | 1 | 371 | 0.264 | -1.20 | 986 | 47 | -2.14 | -2.18 |
| *Prosopis velutina* | Fabaceae | 1 | 132 | 0.258 | -1.92 | 1024 | 123 | 1.23 | 0.88 |
| *Robinia neomexicana* | Fabaceae | 1 | 280 | 0.711 | 1.37 | 899 | 114 | 0.05 | 0.17 |
| *Robinia pseudoacacia* | Fabaceae | 1 | 1389 | 0.601 | -1.49 | 14936 | 48 | -0.93 | -0.40 |
| *Acer barbatum* | Sapindaceae | 0 | 1033 | 0.963 | 0.92 | 2604 | 52 | 0.66 | 0.75 |
| *Acer glabrum* | Sapindaceae | 0 | 841 | 0.765 | 0.25 | 2201 | 79 | -0.01 | -1.24 |
| *Acer grandidentatum* | Sapindaceae | 0 | 691 | 0.588 | 1.05 | 3394 | 63 | -1.54 | -2.41 |
| *Acer leucoderme* | Sapindaceae | 0 | 11 |  |  | 54 | 44 | 0.05 | -0.33 |
| *Acer macrophyllum* | Sapindaceae | 0 | 114 | 0.719 | 0.00 | 1783 | 92 | -0.87 | -1.31 |
| *Acer negundo* | Sapindaceae | 0 | 3959 | 0.839 | 0.36 | 16945 | 52 | -0.36 | -0.30 |
| *Acer nigrum* | Sapindaceae | 0 | 79 | 1.000 | 0.86 | 365 | 65 | 0.38 | 1.27 |
| *Acer pensylvanicum* | Sapindaceae | 0 | 3537 | 0.877 | 0.61 | 7539 | 69 | 0.11 | 0.98 |
| *Acer rubrum* | Sapindaceae | 0 | 94437 | 0.858 | 0.53 | 426395 | 60 | 0.23 | 0.26 |
| *Acer saccharinum* | Sapindaceae | 0 | 1630 | 0.829 | 0.18 | 14966 | 60 | 0.16 | 0.14 |
| *Acer saccharum* | Sapindaceae | 0 | 48246 | 0.933 | 1.20 | 269935 | 70 | 0.66 | 0.29 |
| *Acer spicatum* | Sapindaceae | 0 | 5046 | 0.923 | 2.03 | 6829 | 68 | 0.37 | 0.06 |
| *Acer* spp. | Sapindaceae | 0 | 10 |  |  | 29 | 48 | -1.72 | -1.00 |
| *Aesculus californica* | Sapindaceae | 0 | 73 | 0.329 | -2.24 | 259 | 72 | -1.66 | -2.18 |
| *Aesculus flava* | Sapindaceae | 0 | 152 | 0.980 | 0.68 | 1482 | 62 | 0.30 | 1.00 |
| *Aesculus glabra* | Sapindaceae | 0 | 419 | 0.990 | 0.88 | 1042 | 58 | -0.18 | -0.04 |
| *Aesculus* spp. | Sapindaceae | 0 | 196 | 0.969 | 0.86 | 1002 | 63 | 0.24 | 1.08 |
| *Amelanchier arborea* | Rosaceae | 0 | 18 |  |  | 29 | 53 | -0.58 | -0.21 |
| *Amelanchier* spp. | Rosaceae | 0 | 1918 | 0.947 | 0.87 | 5383 | 62 | 0.01 | 0.95 |
| *Arbutus menziesii* | Ericaceae | 0 | 163 | 0.644 | -0.61 | 2480 | 98 | -0.57 | -1.35 |
| *Asimina triloba* | Annonaceae | 0 | 752 | 0.964 | 0.70 | 1195 | 58 | -0.11 | 0.32 |
| *Betula alleghaniensis* | Betulaceae | 0 | 5783 | 0.829 | 0.49 | 49164 | 76 | 0.89 | 0.75 |
| *Betula lenta* | Betulaceae | 0 | 1945 | 0.806 | -0.20 | 21106 | 67 | 0.19 | 1.49 |
| *Betula nigra* | Betulaceae | 0 | 415 | 0.682 | -0.90 | 3699 | 49 | -0.18 | -0.10 |
| *Betula papyrifera* | Betulaceae | 0 | 19882 | 0.649 | -0.17 | 118212 | 58 | -0.03 | -0.75 |
| *Betula populifolia* | Betulaceae | 0 | 795 | 0.426 | -2.30 | 3582 | 46 | -1.48 | -0.41 |
| *Betula* spp. | Betulaceae | 0 | 1066 | 0.815 | -0.18 | 4364 | 61 | 0.64 | 0.44 |
| *Carpinus caroliniana* | Betulaceae | 0 | 13321 | 0.959 | 1.03 | 26967 | 53 | 0.49 | 0.44 |
| *Carya alba* | Juglandaceae | 0 | 4659 | 0.908 | 0.42 | 25186 | 58 | 0.39 | 0.52 |
| *Carya aquatica* | Juglandaceae | 0 | 151 | 0.868 | 0.28 | 1161 | 59 | 1.47 | 1.64 |
| *Carya cordiformis* | Juglandaceae | 0 | 2586 | 0.872 | 0.38 | 12481 | 59 | 0.01 | 0.25 |
| *Carya glabra* | Juglandaceae | 0 | 4794 | 0.931 | 0.53 | 27010 | 63 | 0.64 | 0.69 |
| *Carya illinoinensis* | Juglandaceae | 0 | 135 | 0.689 | -0.71 | 1184 | 48 | -0.08 | 0.08 |
| *Carya laciniosa* | Juglandaceae | 0 | 114 | 0.868 | 0.17 | 1148 | 62 | 0.46 | 0.48 |
| *Carya myristiciformis* | Juglandaceae | 0 | 1 |  |  | 22 | 51 | 1.17 | 2.37 |
| *Carya ovata* | Juglandaceae | 0 | 3757 | 0.908 | 0.51 | 23316 | 63 | 0.38 | 0.47 |
| *Carya pallida* | Juglandaceae | 0 | 43 | 0.767 | -0.28 | 262 | 54 | 0.48 | 0.54 |
| *Carya* spp. | Juglandaceae | 0 | 6699 | 0.926 | 0.59 | 32951 | 57 | 0.44 | 0.57 |
| *Carya texana* | Juglandaceae | 0 | 4010 | 0.903 | 0.41 | 12244 | 57 | 0.02 | -0.17 |
| *Castanea dentata* | Fagaceae | 0 | 337 | 0.902 | 0.23 | 627 | 66 | 0.32 | 0.86 |
| *Castanea pumila* | Fagaceae | 0 | 11 |  |  | 25 | 36 | -1.04 | -0.97 |
| *Catalpa bignonioides* | Bignoniaceae | 0 | 7 |  |  | 41 | 40 | -0.37 | -0.07 |
| *Catalpa speciosa* | Bignoniaceae | 0 | 40 | 0.975 | 0.72 | 168 | 55 | -0.15 | 0.50 |
| *Catalpa* spp. | Bignoniaceae | 0 | 3 |  |  | 84 | 55 | -0.13 | 0.97 |
| *Celtis laevigata* | Cannabaceae | 0 | 598 | 0.923 | 0.54 | 4476 | 51 | 0.52 | 0.94 |
| *Celtis occidentalis* | Cannabaceae | 0 | 3809 | 0.928 | 0.68 | 15689 | 54 | -0.05 | 0.25 |
| *Celtis* spp. | Cannabaceae | 0 | 417 | 0.945 | 0.90 | 1450 | 61 | 1.89 | 1.17 |
| *Cercis canadensis* | Fabaceae | 0 | 4749 | 0.903 | 0.40 | 8766 | 48 | -0.68 | -0.44 |
| *Chrysolepis chrysophylla* var. *chrysophylla* | Fagaceae | 0 | 103 | 0.796 | 0.22 | 653 | 120 | 0.29 | -0.79 |
| *Cornus florida* | Cornaceae | 0 | 33081 | 0.965 | 0.77 | 56211 | 49 | -0.27 | -0.32 |
| *Cornus nuttallii* | Cornaceae | 0 | 92 | 0.880 | 1.38 | 190 | 143 | 1.19 | -0.11 |
| *Crataegus crus-galli* | Rosaceae | 0 | 47 | 1.000 | 1.25 | 98 | 52 | -0.35 | -0.40 |
| *Crataegus mollis* | Rosaceae | 0 | 20 | 0.850 | 0.26 | 55 | 43 | -1.30 | -0.82 |
| *Crataegus* spp. | Rosaceae | 0 | 4182 | 0.832 | 0.21 | 8296 | 40 | -1.49 | -0.70 |
| *Diospyros virginiana* | Ebenaceae | 0 | 4004 | 0.773 | -0.30 | 9227 | 41 | -0.79 | -0.83 |
| *Fagus grandifolia* | Fagaceae | 0 | 10200 | 0.954 | 0.95 | 69612 | 70 | 0.68 | 1.24 |
| *Fraxinus americana* | Oleaceae | 0 | 7873 | 0.838 | 0.22 | 55404 | 60 | -0.02 | 0.41 |
| *Fraxinus caroliniana* | Oleaceae | 0 | 68 | 0.882 | 0.75 | 201 | 55 | 0.78 | 1.13 |
| *Fraxinus latifolia* | Oleaceae | 0 | 6 |  |  | 70 | 65 | -1.60 | -1.66 |
| *Fraxinus nigra* | Oleaceae | 0 | 23166 | 0.772 | 0.75 | 71161 | 66 | 0.80 | -0.36 |
| *Fraxinus pennsylvanica* | Oleaceae | 0 | 9913 | 0.820 | 0.46 | 42163 | 56 | 0.21 | 0.16 |
| *Fraxinus profunda* | Oleaceae | 0 | 76 | 0.908 | 0.66 | 380 | 74 | 3.16 | 2.16 |
| *Fraxinus quadrangulata* | Oleaceae | 0 | 127 | 0.866 | 0.07 | 568 | 56 | -0.11 | 0.06 |
| *Fraxinus* spp. | Oleaceae | 0 | 8029 | 0.932 | 0.93 | 17799 | 63 | 1.66 | 1.27 |
| *Gleditsia aquatica* | Fabaceae | 0 | 15 |  |  | 139 | 55 | 0.71 | 0.74 |
| *Gleditsia triacanthos* | Fabaceae | 0 | 1020 | 0.582 | -1.46 | 5902 | 46 | -0.83 | -0.51 |
| *Gordonia lasianthus* | Theaceae | 0 | 3063 | 0.724 | -0.30 | 7357 | 48 | 0.17 | 0.09 |
| *Gymnocladus dioicus* | Fabaceae | 0 | 107 | 0.850 | 0.30 | 525 | 54 | -0.07 | 0.26 |
| *Halesia* spp. | Styracaceae | 0 | 200 | 0.950 | 0.45 | 682 | 75 | 1.29 | 0.65 |
| *Ilex opaca* | Aquifoliaceae | 0 | 7776 | 0.981 | 0.98 | 16530 | 51 | 0.48 | 0.41 |
| *Juglans cinerea* | Juglandaceae | 0 | 138 | 0.739 | -0.37 | 1832 | 57 | -0.22 | -0.24 |
| *Juglans hindsii* | Juglandaceae | 0 | 1 |  |  | 59 | 80 | -0.79 | 0.21 |
| *Juglans major* | Juglandaceae | 0 | 0 |  |  | 21 | 95 | -0.75 | -0.46 |
| *Juglans nigra* | Juglandaceae | 0 | 1544 | 0.760 | -0.38 | 17150 | 52 | -0.45 | -0.16 |
| *Juglans* spp. | Juglandaceae | 0 | 2 |  |  | 79 | 84 | -1.13 | -1.04 |
| *Laguncularia racemosa* | Combretaceae | 0 | 28 | 0.429 | -1.69 | 0 |  |  |  |
| *Liquidambar styraciflua* | Altingiaceae | 0 | 34761 | 0.840 | 0.17 | 145699 | 45 | 0.04 | -0.15 |
| *Liriodendron tulipifera* | Magnoliaceae | 0 | 9616 | 0.778 | -0.36 | 94193 | 52 | -0.11 | 0.02 |
| *Lithocarpus densiflorus* | Fagaceae | 0 | 758 | 0.826 | 0.49 | 5419 | 111 | -0.09 | -1.04 |
| *Maclura pomifera* | Moraceae | 0 | 1379 | 0.740 | -0.44 | 6017 | 40 | -1.28 | -0.90 |
| *Magnolia acuminate* | Magnoliaceae | 0 | 276 | 0.964 | 0.63 | 3177 | 63 | 0.14 | 1.15 |
| *Magnolia fraseri* | Magnoliaceae | 0 | 124 | 0.911 | 0.26 | 1002 | 67 | 0.61 | 0.46 |
| *Magnolia grandiflora* | Magnoliaceae | 0 | 667 | 0.958 | 1.01 | 2340 | 58 | 1.18 | 1.07 |
| *Magnolia macrophylla* | Magnoliaceae | 0 | 118 | 0.924 | 0.56 | 431 | 57 | 1.24 | 1.41 |
| *Magnolia* spp. | Magnoliaceae | 0 | 14 |  |  | 471 | 60 | -0.28 | 1.01 |
| *Magnolia virginiana* | Magnoliaceae | 0 | 9758 | 0.841 | 0.37 | 23600 | 53 | 0.86 | 0.72 |
| *Malus fusca* | Rosaceae | 0 | 22 | 0.136 | -4.07 | 33 | 51 | -2.91 | -2.67 |
| *Malus* spp. | Rosaceae | 0 | 793 | 0.784 | -0.12 | 4263 | 38 | -1.79 | -0.85 |
| *Morus rubra* | Moraceae | 0 | 1377 | 0.914 | 0.55 | 4250 | 51 | -0.28 | 0.00 |
| *Morus* spp. | Moraceae | 0 | 250 | 0.940 | 0.78 | 639 | 52 | 0.59 | 0.34 |
| *Nyssa aquatica* | Nyssaceae | 0 | 1424 | 0.929 | 0.84 | 16696 | 76 | 3.49 | 2.53 |
| *Nyssa biflora* | Nyssaceae | 0 | 19581 | 0.867 | 0.56 | 73641 | 61 | 1.82 | 1.14 |
| *Nyssa ogeche* | Nyssaceae | 0 | 367 | 0.883 | 0.70 | 1392 | 64 | 2.22 | 1.61 |
| *Nyssa sylvatica* | Nyssaceae | 0 | 15339 | 0.956 | 0.67 | 46383 | 57 | 0.36 | 0.66 |
| *Ostrya virginiana* | Betulaceae | 0 | 16910 | 0.925 | 1.06 | 35515 | 62 | 0.31 | 0.28 |
| *Oxydendrum arboretum* | Ericaceae | 0 | 11148 | 0.952 | 0.64 | 34025 | 56 | 0.32 | 0.27 |
| *Persea borbonia* | Lauraceae | 0 | 5777 | 0.854 | 0.43 | 9925 | 51 | 0.59 | 0.22 |
| *Planera aquatica* | Ulmaceae | 0 | 167 | 0.886 | 0.48 | 701 | 58 | 1.32 | 1.22 |
| *Platanus occidentalis* | Platanaceae | 0 | 715 | 0.662 | -1.05 | 9923 | 53 | -0.08 | -0.06 |
| *Populus angustifolia* | Salicaceae | 0 | 27 | 0.481 | 0.54 | 507 | 94 | -0.63 | -0.78 |
| *Populus balsamifera* | Salicaceae | 0 | 8496 | 0.487 | -1.20 | 36499 | 50 | -0.51 | -1.52 |
| *Populus balsamifera* sub. *trichocarpa* | Salicaceae | 0 | 50 | 0.660 | -0.54 | 595 | 75 | -1.68 | -1.78 |
| *Populus deltoides* | Salicaceae | 0 | 464 | 0.567 | -1.44 | 6381 | 53 | -0.45 | 0.13 |
| *Populus deltoides* sub. *monilifera* | Salicaceae | 0 | 7 |  |  | 329 | 85 | -0.61 | 0.03 |
| *Populus fremontii* | Salicaceae | 0 | 1 |  |  | 149 | 102 | -0.65 | -0.87 |
| *Populus grandidentata* | Salicaceae | 0 | 10345 | 0.529 | -1.30 | 59850 | 54 | -0.28 | -0.83 |
| *Populus heterophylla* | Salicaceae | 0 | 12 |  |  | 186 | 60 | 1.59 | 0.68 |
| *Populus* spp. | Salicaceae | 0 | 90 | 0.733 | -0.36 | 589 | 63 | 1.33 | 0.68 |
| *Populus tremuloides* | Salicaceae | 0 | 81317 | 0.490 | -1.27 | 338717 | 52 | -0.80 | -1.48 |
| *Prunus americana* | Rosaceae | 0 | 486 | 0.844 | 0.23 | 642 | 44 | -1.27 | -1.21 |
| *Prunus emarginata* | Rosaceae | 0 | 33 | 0.333 | -2.78 | 100 | 53 | -1.46 | -2.28 |
| *Prunus pennsylvanica* | Rosaceae | 0 | 1899 | 0.603 | -0.82 | 4267 | 42 | -1.63 | -1.09 |
| *Prunus serotina* | Rosaceae | 0 | 16061 | 0.788 | 0.00 | 76109 | 52 | -0.59 | 0.02 |
| *Prunus* spp. | Rosaceae | 0 | 170 | 0.859 | 0.82 | 690 | 50 | -0.33 | -0.08 |
| *Prunus virginiana* | Rosaceae | 0 | 2218 | 0.851 | 1.09 | 3329 | 45 | -1.13 | -1.21 |
| *Quercus agrifolia* | Fagaceae | 0 | 21 | 0.571 | 0.37 | 606 | 110 | 0.03 | -0.79 |
| *Quercus alba* | Fagaceae | 0 | 15612 | 0.893 | 0.41 | 159654 | 66 | 0.82 | 0.73 |
| *Quercus arizonica* | Fagaceae | 0 | 1203 | 0.482 | -0.49 | 7393 | 118 | 0.10 | 0.11 |
| *Quercus bicolor* | Fagaceae | 0 | 206 | 0.893 | 0.69 | 2686 | 64 | 0.48 | 0.86 |
| *Quercus buckleyi* | Fagaceae | 0 | 75 | 0.840 | 0.07 | 573 | 57 | 1.02 | 1.33 |
| *Quercus chrysolepis* | Fagaceae | 0 | 915 | 0.597 | -0.34 | 7410 | 106 | -0.17 | -1.05 |
| *Quercus coccinea* | Fagaceae | 0 | 2598 | 0.748 | -0.59 | 34831 | 61 | 0.39 | 0.57 |
| *Quercus douglasii* | Fagaceae | 0 | 56 | 0.232 | -3.00 | 1136 | 82 | -1.00 | -1.94 |
| *Quercus ellipsoidalis* | Fagaceae | 0 | 1083 | 0.763 | 0.22 | 10756 | 57 | -0.09 | -0.43 |
| *Quercus emoryi* | Fagaceae | 0 | 344 | 0.669 | 1.19 | 2718 | 127 | 0.68 | 0.72 |
| *Quercus* spp. (evergreen) | Fagaceae | 0 | 669 | 0.167 | -3.16 | 4769 | 93 | -0.89 | -0.86 |
| *Quercus falcata* | Fagaceae | 0 | 4180 | 0.798 | -0.15 | 28706 | 48 | 0.13 | 0.13 |
| *Quercus gambelii* | Fagaceae | 0 | 18337 | 0.402 | -0.92 | 37860 | 90 | -0.96 | -1.14 |
| *Quercus garryana* | Fagaceae | 0 | 143 | 0.455 | -1.40 | 1698 | 81 | -1.30 | -1.62 |
| *Quercus hypoleucoides* | Fagaceae | 0 | 498 | 0.357 | -1.22 | 1358 | 126 | 0.54 | 0.83 |
| *Quercus ilicifolia* | Fagaceae | 0 | 87 | 0.713 | -0.72 | 219 | 51 | -0.86 | 0.54 |
| *Quercus imbricaria* | Fagaceae | 0 | 830 | 0.764 | -0.45 | 3722 | 49 | -0.81 | -0.76 |
| *Quercus incana* | Fagaceae | 0 | 1511 | 0.708 | -0.47 | 2550 | 38 | -0.58 | -0.66 |
| *Quercus kelloggii* | Fagaceae | 0 | 261 | 0.548 | -0.75 | 4390 | 88 | -0.75 | -1.67 |
| *Quercus laevis* | Fagaceae | 0 | 4976 | 0.565 | -1.37 | 8585 | 41 | -0.41 | -0.42 |
| *Quercus laurifolia* | Fagaceae | 0 | 7364 | 0.838 | 0.40 | 30124 | 52 | 0.96 | 0.57 |
| *Quercus lobata* | Fagaceae | 0 | 2 |  |  | 141 | 111 | 0.48 | -1.05 |
| *Quercus lyrata* | Fagaceae | 0 | 283 | 0.905 | 0.62 | 3048 | 64 | 2.07 | 1.78 |
| *Quercus macrocarpa* | Fagaceae | 0 | 4179 | 0.837 | 1.24 | 30613 | 64 | 0.50 | 0.16 |
| *Quercus margarettiae* | Fagaceae | 0 | 258 | 0.744 | -0.30 | 710 | 37 | -0.55 | -0.76 |
| *Quercus marilandica* | Fagaceae | 0 | 3511 | 0.756 | -0.40 | 10154 | 47 | -0.37 | -0.73 |
| *Quercus michauxii* | Fagaceae | 0 | 501 | 0.944 | 0.84 | 3278 | 57 | 1.32 | 0.91 |
| *Quercus minima* | Fagaceae | 0 | 47 | 0.596 | -1.01 | 271 | 41 | -0.73 | -0.17 |
| *Quercus muehlenbergii* | Fagaceae | 0 | 1203 | 0.847 | 0.12 | 9195 | 62 | 0.42 | 0.25 |
| *Quercus nigra* | Fagaceae | 0 | 14701 | 0.785 | -0.09 | 55187 | 41 | -0.15 | -0.27 |
| *Quercus oblongifolia* | Fagaceae | 0 | 9 |  |  | 237 | 150 | 3.48 | 2.17 |
| *Quercus pagoda* | Fagaceae | 0 | 327 | 0.810 | -0.07 | 4017 | 52 | 0.77 | 0.84 |
| *Quercus palustris* | Fagaceae | 0 | 301 | 0.764 | -0.34 | 3585 | 54 | -0.25 | 0.08 |
| *Quercus phellos* | Fagaceae | 0 | 1779 | 0.797 | -0.11 | 10398 | 49 | 0.49 | 0.32 |
| *Quercus prinus* | Fagaceae | 0 | 3271 | 0.846 | -0.05 | 66656 | 71 | 0.93 | 1.53 |
| *Quercus rubra* | Fagaceae | 0 | 8801 | 0.809 | 0.34 | 130123 | 68 | 0.69 | 0.62 |
| *Quercus rugosa* | Fagaceae | 0 | 32 | 0.688 | 2.24 | 79 | 93 | -0.88 | -0.63 |
| *Quercus shumardii* | Fagaceae | 0 | 85 | 0.788 | -0.21 | 1188 | 55 | 0.56 | 0.50 |
| *Quercus similis* | Fagaceae | 0 | 1 |  |  | 26 | 43 | -0.77 | -0.13 |
| *Quercus sinuata* var. *sinuata* | Fagaceae | 0 | 5 |  |  | 54 | 51 | 1.09 | 1.10 |
| *Quercus* spp. | Fagaceae | 0 | 2966 | 0.615 | -0.91 | 4364 | 39 | -0.79 | -0.62 |
| *Quercus stellata* | Fagaceae | 0 | 7744 | 0.801 | -0.15 | 49035 | 56 | 0.33 | -0.04 |
| *Quercus velutina* | Fagaceae | 0 | 9264 | 0.797 | -0.18 | 79928 | 62 | 0.32 | 0.31 |
| *Quercus virginiana* | Fagaceae | 0 | 2104 | 0.713 | -0.19 | 9339 | 56 | 0.92 | 1.05 |
| *Quercus wislizeni* | Fagaceae | 0 | 362 | 0.425 | -1.53 | 1366 | 73 | -1.53 | -2.05 |
| *Sabal palmetto* | Arecaceae | 0 | 5 |  |  | 1055 | 49 | -0.25 | 0.49 |
| *Salix amygdaloides* | Salicaceae | 0 | 251 | 0.789 | 0.90 | 539 | 48 | -0.87 | -0.97 |
| *Salix bebbiana* | Salicaceae | 0 | 209 | 0.656 | -0.59 | 267 | 40 | -1.52 | -2.01 |
| *Salix nigra* | Salicaceae | 0 | 880 | 0.486 | -2.00 | 5982 | 47 | -0.81 | -0.36 |
| *Salix* spp. | Salicaceae | 0 | 908 | 0.564 | -1.22 | 3451 | 37 | -1.11 | -1.08 |
| *Sassafras albidum* | Lauraceae | 0 | 8393 | 0.878 | 0.22 | 26347 | 52 | -0.53 | -0.08 |
| *Sideroxylon lanuginosum* sub. *lanuginosum* | Sapotaceae | 0 | 134 | 0.948 | 0.70 | 244 | 57 | 0.40 | 0.16 |
| *Sorbus americana* | Rosaceae | 0 | 257 | 0.665 | -0.50 | 655 | 56 | -0.86 | -0.12 |
| *Tilia americana* | Malvaceae | 0 | 6822 | 0.906 | 1.31 | 62070 | 65 | 0.48 | -0.11 |
| *Tilia americana* var. *caroliniana* | Malvaceae | 0 | 6 |  |  | 79 | 65 | 2.24 | 2.39 |
| *Tilia americana* var. *heterophylla* | Malvaceae | 0 | 36 | 0.972 | 0.83 | 296 | 66 | 1.32 | 1.36 |
| *Tilia* spp. | Malvaceae | 0 | 237 | 0.954 | 0.65 | 1313 | 67 | 0.70 | 1.04 |
| *Ulmus alata* | Ulmaceae | 0 | 4441 | 0.918 | 0.48 | 14653 | 47 | -0.10 | 0.29 |
| *Ulmus americana* | Ulmaceae | 0 | 19177 | 0.889 | 0.70 | 67472 | 54 | -0.26 | -0.41 |
| *Ulmus crassifolia* | Ulmaceae | 0 | 51 | 0.922 | 0.53 | 455 | 58 | 1.34 | 2.01 |
| *Ulmus rubra* | Ulmaceae | 0 | 5643 | 0.907 | 0.49 | 17958 | 57 | -0.11 | 0.16 |
| *Ulmus serotina* | Ulmaceae | 0 | 13 |  |  | 71 | 33 | -0.88 | -0.63 |
| *Ulmus* spp. | Ulmaceae | 0 | 3539 | 0.930 | 0.77 | 9526 | 52 | 0.65 | 0.28 |
| *Ulmus thomasii* | Ulmaceae | 0 | 425 | 0.918 | 0.92 | 1016 | 58 | -0.03 | -0.44 |
| *Umbellularia californica* | Lauraceae | 0 | 207 | 0.749 | 1.04 | 956 | 84 | -0.81 | -1.49 |

^*^ ‘N fixer’ indicates whether a species is an N fixer (1) or not (0), ‘# *ST*’ is the number of FIA individuals available for calculating *ST* indices (live saplings with reported crown class), ‘*ST_U_*’ is the unweighted shade tolerance index (proportion of saplings in understory; unitless), ‘*ST_W_*’ is the geographically weighted shade tolerance index, ‘# *SA*’ is the number of FIA individuals available for calculating *SA* indices (live saplings + trees in plots with reported stand age), ‘*SA_U_*’ is the geographically-unweighted, basal area-weighted mean stand age in which the species occurs, and ‘*SA_W-mean_*’ and ‘*SA_W-max_*’ are geographically weighted stand age indices (see Methods for details). All weighted indices are normalized within angiosperms to have mean 0 and variance 1. Values are reported only for species with ≥ 20 individuals. The data are organized by N-fixing status, then alphabetically by genus and species.
